# Structural Variations of Tendons: A Systematic Search and Narrative Review of Histological Differences Between Tendons, Tendon Regions, Sex, and Age

**DOI:** 10.1002/jor.26060

**Published:** 2025-02-26

**Authors:** Samantha A. Hefferan, Carina L. Blaker, Dylan M. Ashton, Christopher B. Little, Elizabeth C. Clarke

**Affiliations:** ^1^ Murray Maxwell Biomechanics Laboratory Institute of Bone and Joint Research, Kolling Institute, Royal North Shore Hospital, Northern Sydney Local Health District Sydney New South Wales Australia; ^2^ School of Medical Sciences, Sydney Musculoskeletal Health, Faculty of Medicine and Health, University of Sydney Sydney New South Wales Australia; ^3^ Sydney School of Veterinary Science, Faculty of Science, University of Sydney Sydney New South Wales Australia; ^4^ Raymond Purves Bone & Joint Research Laboratories Institute of Bone and Joint Research, Kolling Institute, Royal North Shore Hospital, Northern Sydney Local Health District Sydney New South Wales Australia

**Keywords:** healthy/normal, ligament, microscopy, tendon, tissue architecture

## Abstract

Tendons are force‐transmitting structures which facilitate musculoskeletal functioning. Characterizing variations between different anatomical tendons, regions within tendons, as well as between the sexes and with age can improve understanding of tendon physiology and pathology. A systematic search of the literature was conducted to identify and summarize microscopic structural (histological) variations in normal/healthy tendons in relation to these variables (Tendon, Region, Age, Sex, and Other). Regional differences within individual tendons have been investigated in numerous studies, however investigations comparing histological variations between a range of different tendons are sparse, with most focusing on a few select tendons. When injured, ageing tendons typically have a greater degree of pathological changes than younger tendons, but few studies have documented variations in tendon histology throughout typical (uninjured) ageing or across large age spans. Similarly, sex‐related observations of tendon structure are underreported. This narrative review summarizes studies on these topics and explores interactions between these variables, as well as the implications of these in the context of selecting control samples for studies of tendon pathology. Future studies should endeavour to improve knowledge of tendon structural variations—specifically focusing on normal tendons—to facilitate understanding of tendon structure‐function relationships, physiological mechanisms involved in tendon damage/healing, and to aid clinical research and practice.

## 1 Introduction

Tendons are force transmitting units that facilitate muscular movement of bone and joints, playing an important role in biomechanical and locomotor function [1] contributing to musculoskeletal and overall well‐being. Different anatomical tendons exhibit different roles and experience varied loading environments. For example, the distinction between “energy‐storing” tendons versus “positional” tendons identifies unique roles for locomotor efficiency in quadrupeds and human/bipedal lower limbs [1, 2, 3, 4, 5]. Conversely, upper limb tendons in humans/bipeds play an important role in finer manipulation/control and may portray different gross morphologies dependent on their anatomical role, such as the rope‐like digital flexors versus the flatter extensors which fan out into aponeurosis‐like structures and exhibit “offshoot slips” between neighboring tendons [6].

Tendons are prone to injury, pathology and degenerative processes [7, 8, 9]. Despite the advent of different imaging modalities, histology remains a gold standard for assessing tissue health and disease [10, 11, 12, 13, 14, 15, 16]. Understanding normal tendon histological features is critical for gaining insight into the mechanisms of tendon damage, disease, degeneration, and repair [17, 18]. While “healthy tendon” histology is often used as a baseline/control for studies of diseased tendons, knowledge about what constitutes “normal” histology and variations in multiscale aspects particularly of human tendons, are poorly explored. This review examines what is known and knowledge gaps on tendon histological characteristics assessed using microscopy modalities, with particular focus on variation between different tendons, tendon regions, ages, and sexes. A systematic literature search was conducted to identify published records which compared multiscale histological structural characteristics of normal/healthy tendons.

## 2 Systematic Literature Search: Structural Variations of Normal Tendons

**Figure 1:**
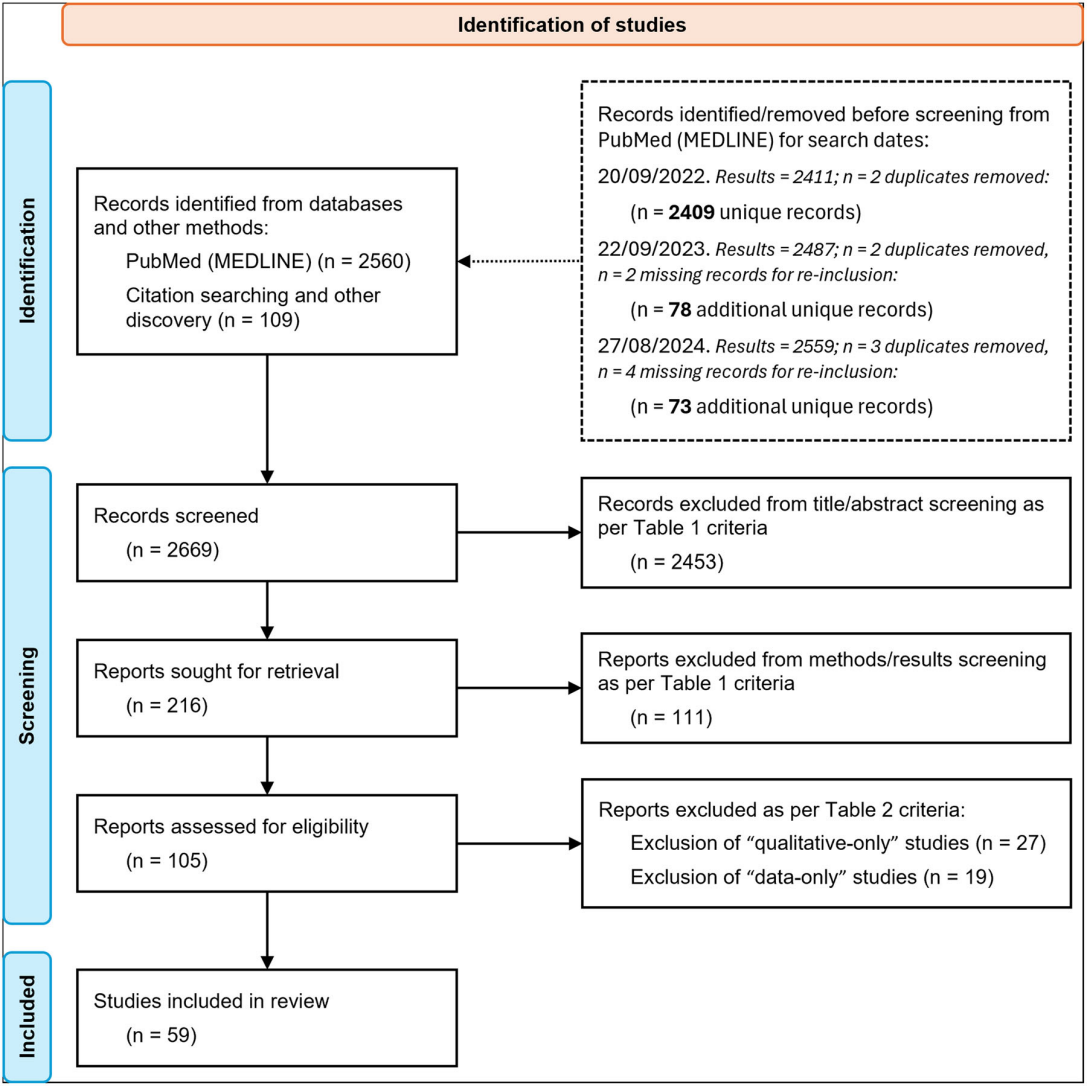
Flow diagram of literature inclusion and screening workflow. Diagram is modified from a readily accessible template [19].

An electronic database search of PubMed (MEDLINE) was conducted with a focus on comparisons between “variables of interest”: *Tendon*; *Region*; *Age*; *Sex*; and *Other*. Keyword search terms were: (tendon OR tendons OR tend*) AND (histolog* OR histochem* OR microscop*) AND (normal OR healthy OR “non‐pathological” OR “non pathological”) AND (structu*) AND (compar* OR differen* OR variation OR variations). Supporting Information S1: Material A contains the full list of generated search terms and MeSH terms, indexed according to the terms list from the National Library of Medicine. The initial search (September 30, 2022) found 2411 records, including 2 duplicate pairs (2409 unique records). The search was re‐run at two subsequent timepoints during manuscript preparation, using the full list of MeSH terms each time to ensure consistency: September 22, 2023 (78 new additional unique articles identified) and August 27, 2024 (73 new additional unique articles identified). A final total of 2560 unique records were identified from the PubMed literature searches (Supporting Information S2: Table 1, Figure 1). Cross‐checking of citations/reference‐lists in the publications found by the systematic search criteria and alternative search strategies/engines (e.g., Google Scholar) identified a further 109 records for review.

All 2669 publications were screened by a single reviewer (S. H.) as per the workflow in Figure 1. Articles were first screened by title and abstract and excluded as per Table 1. The 216 articles passing title/abstract screening had the full text assessed for exclusion/eligibility as per Table 1 criteria. The 105 eligible papers underwent final review according to the criteria in Table 2 to remove publications with “qualitative‐only” (*n* = 27) or “data‐only” (*n* = 19) comparisons (these are recorded/summarized in Supporting Information S2: Table 1 for additional reference).

**Table 1:** Eligibility and exclusion criteria for systematic search strategy (screening stages).

**Exclusion: Criterion for the exclusion of studies from further screening**
Nonprimary research (e.g., reviews, letters, editorials, etc.)
Full text not available through open access, institution subscriptions/resources or not written in English.
Studies *isolated* to any of the following: ‐ Ligaments or ligament comparisons. ‐ Tendon macro‐structure characterization (e.g., anatomical morphology, macroscopic exploration, etc.). ‐ Characterization of non‐appendicular tendons (e.g., spinal, cranial, ocular, auditory, respiratory, etc.). ‐ Enthesis (tendon‐bone junction), musculotendinous junction, aponeurosis, or tendon sheath (epitenon) characterization. ‐ Utilization of medical imaging techniques (e.g., MRI or ultrasound) for structural assessment. **Eligibility: Criterion for considering studies for assessment.** a. Types of studies Primary research studies addressing the comparison of normal/healthy tendon micro‐structure, including pathological/other studies offering comparative analysis of normal/healthy tendons utilized as control tissue Full manuscript text available through open access or institution resources and written in English b. Types of tissue explored Tendons of the appendicular system of any species, sex, or age (excluding prenatal) Tendon mid‐substance (i.e., regions apart from enthesis (tendon‐bone junction) or musculotendinous junction) c. Types of outcome measures Utilization of microscopy techniques (e.g., variants of optical microscopy, electron microscopy, atomic force microscopy, etc.) At least one of the following “variables of interest” explored regarding structural comparisons of normal/healthy tendon mid‐substance specimens: i. *Tendon*: Differences between tendons (or tendons and ligaments) ii. *Region*: Differences between tendon/ligament mid‐substance regions iii. *Age*: Differences in relation to age iv. *Sex*: Differences between males and females v. *Other*: Other identified differences comparing states of “normal” tendon, for example, lifestyle differences (exercise, diet, etc.), species differences, and so forth.

**Table 2:** Final inclusion criteria for publications to be reviewed. Excluded records (“noted exclusions”) remain identified in Supporting Information S2: Table 1 as additional supplementary knowledge explored in the field.

*Additional exclusion criterion A: Exclusion of “qualitative‐only” studies
Studies which solely reported qualitative or descriptive comparisons for the “variables of interest”
* A list of these publications (n = 27) can be found in Supporting Information S2: Table 1 (“Noted Exclusions A”)
**Additional exclusion criterion B: Exclusions of “data‐only” studies
Studies which conducted objective measures in relation to any of the “variables of interest” but did not explicitly report statistical comparisons for these variables
** These publications (*n* = 19) are summarized and can be found in Supporting Information S2: Table 1 (“Noted Exclusions B”)
**Inclusions: Criterion for inclusion in summary Table** 3
Studies which provided objective comparisons and reported statistical analysis for at least one of the variables of interest (Tendon, Region, Age, Sex, or Other).

## 3 Results

A total of 59 records representing studies which explicitly performed quantitative statistical comparisons for the “variables of interest” were included and summarized in Tables 3 and 4. Table 5 outlines acronyms referred to in this manuscript, and the Tables and Supporting Information Tables.

**Table 3:** Summary of papers exploring statistical comparisons relating to *Tendon*, *Region*, *Age*, *Sex*, or *Other* “variables of interest.” Tendons/Ligaments (T/L) included in the study but not statistically assessed for any of the variables are grayed‐out. Y = statistical analysis is included for the relevant variable. D = data/graphical representation of data is included for the relevant variable.

References	Species	Tendons (T) | Ligaments (L)		Microscopy modality	Tendon	Region	Age	Sex	Other
Ingelmark [20]	Murine (Rat)	1T	Achilles	TEM	—	—	**Y**	—	**Y**
Wilmink et al. [21]	Equine	1T	SDFT	OM	—	**Y**	**Y**	—	—
Patterson‐Kane et al. [22]	Equine	1T	SDFT	OM, TEM	—	**Y**	**Y**	—	—
Patterson‐Kane et al. [23]	Equine	1T	SDFT	OM	—	**Y**	D	—	D
Birch et al. [24]	Equine	2T	DDFT, SDFT	TEM	**Y**	**Y**	**Y**	—	—
Odetti et al. [25]	Murine (Rat)	1T	Tail	SFM	—	—	—	—	**Y**
Gibson et al. [26]	Equine	1T	SDFT	OM, TEM	—	**Y**	—	—	—
Cook et al. [27]	Human	1T	Patellar	OM	—	**Y**	**Y**	—	**Y**
Edwards et al. [28]	Equine	1T	CDET	TEM	—	—	**Y**	—	**Y**
Watanabe et al. [29]	Equine	1T	SDFT	TEM, hvEM	—	**Y**	—	—	—
Prado et al. [30]	Human	1T	Tibialis Posterior	OM	—	**Y**	**Y**	**Y**	**Y**
Hadjicostas et al. [31]	Human	2T	Patellar, Quadriceps	OM, TEM	**Y**	—	**Y**	**Y**	—
Hadjicostas et al. [32]	Human	3T	Gracilis, Semitendinosus, Patellar	OM, TEM	**Y**	—	**Y**	**Y**	—
Funakoshi et al. [33]	Caprine	2T	Infraspinatus, Patellar	OM	—	**Y**	—	—	**Y**
Hadjicostas et al. [34]	Human	4T|1L	Gracilis, Semitendinosus, Patellar, Quadriceps | ACL	OM, TEM	**Y**	—	**Y**	**Y**	—
Hadjicostas et al. [35]	Human	2T	Gracilis, Semitendinosus	OM, TEM	**Y**	—	—	—	—
Stanley et al. [36]	Equine	2T	CDET, SDFT	OM	**Y**	**Y**	D	—	**Y**
Williams et al. [37]	Leporine (Rabbit)	1T	Patellar	TEM	—	**Y**	—	—	—
Franchi et al. [38]	Murine (Rat)	3T	Patellar, Rectus Femoris, Vastus Intermedius	OM	**Y**	**Y**	—	—	—
Hansen et al. [39]	Human	1T	Patellar	TEM	—	—	—	—	**Y**
Hansen et al. [40]	Human	1T	Patellar	TEM	—	**Y**	—	—	—
Hosaka et al. [41]	Equine	3T	CDET, DDFT, SDFT	OM	**Y**	—	—	—	—
Frizziero et al. [42]	Murine (Rat)	1T	Patellar	OM	—	—	—	—	**Y**
Hu et al. [43]	Murine (Rat)	1T	*Unnamed*	SHG	—	—	**Y**	—	—
Weiss et al. [44]	Human	5T|1L	Gracilis, Semitendinosus, Patellar, Quadriceps, Tibialis Anterior | ACL	OM	**Y**	—	—	—	—
Zhu et al. [45]	Human	*1T|1L	“Hamstring” (*Gracilis or Semitendinosus)|ACL	SEM, TEM	**Y**	—	—	—	—
Dunkman et al. [46]	Murine (Mouse)	1T	Patellar	OM, TEM	—	—	**Y**	—	—
Lavagnino et al. [47]	Murine (Rat)	1T	Tail	OM	—	—	**Y**	—	—
Mazzocca et al. [48]	Human	1T	LHBT	OM	—	**Y**	—	—	—
Thorpe et al. [49]	Equine	1T	SDFT	SEM	—	—	**Y**	—	—
Legerlotz et al. [50]	Murine (Mouse)	1T	Tail	OM	—	—	**Y**	—	—
Tilley et al. [51]	Human	2T	Subscapularis, Supraspinatus	OM, AFM	**Y**	—	**Y**	—	—
Lenskjold et al. [52]	Human	1T	Achilles	TEM	—	—	—	—	**Y**
Herod et al. [53]	Bovine	2T	CDET, SDFT	SEM	**Y**	—	—	—	—
Pardes et al. [54]	Murine (Rat)	1T	Achilles	OM	—	—	—	**Y**	**Y**
Thorpe et al. [55]	Equine	2T	CDET, SDFT	OM	**Y**	**Y**	—	—	—
Thorpe et al. [56]	Equine	1T	SDFT	OM	—	**Y**	**Y**	—	—
Biasutti et al. [57]	Ovine	1T	SDFT	OM	—	**Y**	—	—	—
Godinho et al. [58]	Equine	2T	CDET, SDFT	OM	**Y**	D	**Y**	—	—
Marquez‐Arabia et al. [59]	Human	1T	Gluteus Medius	OM	—	—	**Y**	—	—
Pardes et al. [60]	Murine (Rat)	1T	Achilles	OM	—	—	**Y**	—	—
Sarver et al. [61]	Murine (Mouse)	1T	Achilles	OM	—	—	—	**Y**	—
Gagliano et al. [62]	Human	2T	Gracilis, Semitendinosus	OM	—	—	**Y**	—	—
Kharaz et al. [63]	Canine	2T|2L	LDET, SDFT|ACL, MCL	OM	**Y**	**Y**	—	—	—
Mazon et al. [64]	Murine (Rat)	1T	Calcaneal (Achilles)	OM	—	—	—	—	**Y**
Shu et al. [65]	Murine (Mouse)	2T	Achilles, Tail	TEM	D	—	**Y**	—	—
Spiesz et al. [66]	Equine	2T	CDET, SDFT	OM	**Y**	**Y**	**Y**	—	—
Takahashi et al. [67]	Bovine	1T	SDFT	OM, TEM	—	**Y**	—	—	—
Feo et al. [68]	Avian (Pedrês chicken)	1T	SDFT	OM	—	**Y**	**Y**	—	—
Hayashi et al. [69]	Canine	1T|3L	LDET | CaCL, CrCL, MCL	OM	**Y**	—	**Y**	**Y**	**Y**
Ristaniemi et al. [70]	Bovine	1T|4L	Patellar | ACL, LCL, MCL, PCL	OM	**Y**	—	—	—	—
Asai et al. [71]	Human	1T	Semitendinosus	OM, TEM	—	—	**Y**	**Y**	**Y**
Bolam et al. [72]	Murine (Rat)	1T	Achilles	OM, TEM	—	—	—	—	**Y**
Naot et al. [73]	Human	1T	Semitendinosus	TEM/SEM	—	—	**Y**	—	—
Fjordbakk and Marques‐Smith [74]	Equine	3T	Medial Patellar, Intermediate Patellar, Lateral Patellar	OM	**Y**	—	**Y**	—	—
Gsell et al. [75]	Bovine, Murine (Rat)	3T	LDET [bovine], SDFT [bovine], Tail [rat]	AFM	**Y**	—	—	—	**Y**
Johnson et al. [76]	Human	4T	Infraspinatus, Subscapularis, Supraspinatus, Teres Minor	OM	D	—	**Y**	—	—
Tinguely et al. [77]	Murine (Rat), Human	2T	Achilles [rat; human], Patellar [rat]	OM	D	—	**Y**	—	D
Zheng et al. [78]	Avian (Turkey)	1T	Gastrocnemius (Achilles)	sPHG	—	**Y**	—	—	—
*N* = 59	*Total variable (Y) count =*				20	22	30	8	16

Abbreviations: ACL = anterior cruciate ligament, CaCL = caudal cruciate ligament, CDET = common digital extensor tendon, CrCL = cranial cruciate ligament, DDFT = deep digital flexor tendon, hvEM = high voltage electron microscopy, IHC = immunohistochemistry, LCL = lateral collateral ligament, LDET = long digital extensor tendon, LHBT = long head of biceps [brachii] tendon, MCL = medial collateral ligament, OM = optical microscopy (e.g., light, polarized, confocal, etc.), PCL = posterior cruciate ligament, pSHG = polarization‐resolved second harmonic generation microscopy, SDFT = superficial digital flexor tendon, SEM = scanning electron microscopy, SFM = scanning force microscopy, SHG = second harmonic generation microscopy, TEM = transmission electron microscopy.

**Table 4:** Summary of papers by variable (statistical comparisons).

Comparisons (variable)	Number of papers	Number of papers by species										Tendons/ligaments
		Human	Equine	Bovine	Canine	Ovine	Caprine	Rat	Mice	Avian	Leporine	
**Tendon** See Supporting Information S3: Table 2a for details	20	7	7	3	2	—	—	2	—	—	—	Tendons = 17 CDET, DDFT, LDET, SDFT, Patellar, Rectus Femoris, Vastus Intermedius, Quadriceps, Gracilis, Semitendinosus, Tibialis Anterior, Subscapularis, Supraspinatus, Medial Patellar Ligament, Intermediate Patellar Ligament, Lateral Patellar Ligament, Tail *Ligaments* = *6* *ACL, LCL, MCL, PCL, CaCL, CrCL*
**Region** See Supporting Information S3: Table 2b for details	22	4	10	1	1	1	1	1	—	2	1	Tendons = 11 CDET, DDFT, LDET, SDFT, Patellar, Rectus Femoris, Vastus Intermedius, Tibialis Posterior, Infraspinatus, LHBT, Achilles/Gastrocnemius
**Age** See Supporting Information S3: Table 2c for details	30	12	9	—	1	—	—	5	3	1	—	Tendons = 19 CDET, DDFT, LDET, SDFT, Patellar, Medial Patellar Ligament, Intermediate Patellar Ligament, Lateral Patellar Ligament, Quadriceps, Gracilis, Semitendinosus, Tibialis Posterior, Gluteus Medius, Infraspinatus, Subscapularis, Supraspinatus, Teres Minor, Achilles, Tail *Ligaments* = *4* *ACL, MCL, CaCL, CrCL*
**Sex** See Supporting Information S3: Table 2d for details	8	5	—	—	1	—	—	1	1	—	—	Tendons = 7 LDET, Patellar, Quadriceps, Gracilis, Semitendinosus, Tibialis Posterior, Achilles *Ligaments* = *4* *ACL, MCL, CaCL, CrCL*
**Other** See Supporting Information S3: Table 2e for details	16	5	2	1	1	—	1	7	—	—	—	Tendons = 9 CDET, LDET, SDFT, Patellar, Semitendinosus, Tibialis Posterior, Infraspinatus, Achilles/Calcaneal, Tail *Ligaments* = *3* *MCL, CaCL, CrCL*

Abbreviations: ACL = anterior cruciate ligament, CaCL = caudal cruciate ligament, CDET = common digital extensor tendon, CrCL = cranial cruciate ligament, DDFT = deep digital flexor tendon, LCL = lateral collateral ligament, LDET = long digital extensor tendon, LHBT = long head of biceps [brachii] tendon, MCL = medial collateral ligament, PCL = posterior cruciate ligament, SDFT = superficial digital flexor tendon.

**Table 5:** List and definition of acronyms included in manuscript and tables.

Tendon/ligament names	
CDET	Common Digital Extensor Tendon
DDFT	Deep Digital Flexor Tendon
LDET	Long Digital Extensor Tendon
SDFT	Superficial Digital Flexor Tendon
LHBT	Long Head of Biceps [brachii] Tendon
RFT	Rectus Femoris Tendon
VIT	Vastus Intermedius Tendon
TA	Tibialis Anterior
TP	Tibialis Posterior
FHL	Flexor Hallucis Longus
ACL	Anterior Cruciate Ligament
LCL	Lateral Collateral Ligament
MCL	Medial Collateral Ligament
PCL	Posterior Cruciate Ligament
CaCL	Caudal Cruciate Ligament
CrCL	Cranial Cruciate Ligament
**Outcomes**	
ECM	Extracellular matrix
GAG	Glycosaminoglycan
IFM	Interfascicular matrix
FM	Fascicular matrix
IHC	Immunohistochemistry
αSMA	Alpha‐smooth muscle actin
CSA	Cross‐sectional area
TB	Toluidine Blue
PSR	Picrosirius Red
**Microscopy Modalities**	
hvEM	High voltage electron microscopy
IHC	Immunohistochemistry
MLM	Multiphoton laser microscopy
OM	Optical microscopy (e.g. light, polarized, confocal, etc.)
pSHG	Polarization‐resolved second harmonic generation microscopy
SEM	Scanning electron microscopy
SFM	Scanning force microscopy
SHG	Second harmonic generation microscopy
TEM	Transmission electron microscopy

Of the species utilized, 19 studies investigated human specimens, 23 large quadrupeds (15 equine, 4 bovine, 2 canine, 1 ovine, 1 caprine), 16 murine (12 rat, 4 mouse), and 3 other (2 avian, 1 leporine [rabbit]). Only two studies included more than one species [75, 77] (Table 3). Only Gsell et al. [75] compared species but even here direct comparability was limited as different anatomical tendons were utilized across species.

In terms of tendons/ligaments studied, 34 publications investigated a single tendon, 14 investigated two different tendons/ligaments, 5 investigated three, 3 investigated four, 2 investigated five, and 1 investigated six. Considering the *variables of interest* (Table 4), 20 studies compared different anatomical tendons/ligaments (Supporting Information S3: Table 2a), 22 studies compared within‐tendon *Region* differences (Supporting Information S3: Table 2b), 30 *Age*‐related differences (Supporting Information S3: Table 2c), 8 *Sex‐*differences (Supporting Information S3: Table 2d), and 16 explored *Other* variables (Supporting Information S3: Table 2e).

## 4 Between‐Tendon Differences

Direct comparison of tendons is limited. Two studies which passed initial eligibility but not second screening, included a large range of tendons but without any quantitative comparisons. Kannus and Jozsa [12] (Supporting Information S2: Table 1; *Noted Exclusions A*) investigated 16 “tendon‐groups” (individual tendons not specified) and presented only descriptive findings, while another study comparing 24 different appendicular tendons [79] focussed on fibrocartilaginous presence and did not report statistical analyses regarding similarities/difference (Supporting Information S2: Table 1; *Noted Exclusions B*).

Most studies with quantitative analysis of normal tendon structure (34/59, Table 3) only investigated a single tendon. Where at least two tendons (or tendon/ligament) were included (*n* = 25), only 20 explored specific *Tendon* differences (Table 4, Supporting Information S3: Table 2a). Of these, three papers incorporated only one tendon and compared this with ligament tissue(s) [45, 69, 70]. Weiss et al. [44] compared five tendons and the anterior cruciate ligament (ACL) but did not explicitly perform direct tendon‐versus‐tendon statistical comparisons and instead reported similarities/differences in correlated variables (e.g., crimp‐frequency vs. cell‐number). Of the 16 papers that made direct tendon‐to‐tendon statistical comparisons, the majority (10/16) compare only two tendons, 5 studies comparing three, and 1 study each comparing four/five tendons (Supporting Information S3: Table 2a).

Most studies evaluating between‐tendon differences use animals (13/20: equine, bovine, canine, rat), and primarily focus on the comparison of energy‐storing versus positional tendons. There are clear differences reported, with extensor/positional tendons generally having lower cell density, larger collagen fibers, larger crimp wavelength, and different protein makeup within the interfascicular and fascicular matrices (IFM and FM, respectively) in comparison to flexor/energy‐storing tendons (Supporting Information S3: Table 2a). Compared with flexors, positional extensors also have overall, a more organized structure, containing relatively less IFM area [58], less randomly distributed elastin fibers [58], and larger collagen fibrils [53, 75], reflected in an overall greater total birefringence in picrosirius red (PSR) stained sections [66].

Only 7/20 *Tendon‐*comparison papers (Supporting Information S3: Table 2a) evaluated *human* tissues, with the majority (6/7) primarily focused on differences between tendons commonly used as ACL‐reconstruction grafts and three making direct comparisons with the ACL. These studies identified notable differences (e.g., collagen type and fibril size, vascularity, cell density) between the Quadriceps and Patellar [31, 34, 44], the hamstring and Patellar [32, 34, 44], and the Gracilis and Semitendinosus tendons [34, 35, 44] (Supporting Information S3: Table 2a). Interestingly, across all studies and species, the ACL has unique characteristics, differing both from tendons [34, 45, 63, 70] and other ligaments [63, 70]. The seventh human study by Tilley et al. [51] investigating disease progression of rotator cuff tendons, included comparisons of the “normal/control” Supraspinatus and Subscapularis tendons. However, different age cohorts (mean 23.3 years for Subscapularis and 74.6 years for Supraspinatus) confounds direct comparisons.

### 4.1 Current Literature Limitations

Studies which histologically explore different tendons generally lack comprehensive exploration into quantitative between‐tendon differences for a broad range of tendons and/or outcomes and are also often limited by inherent challenges due to the nature of the studies. The limited number of studies and often low sample size (one to three donors [44, 45, 80]), reflect challenges in obtaining human specimens. A general limitation of current microscopy‐based studies is the largely descriptive nature of the reports. For example, observations of collagenous structure commonly include descriptions such as “homogenous,” “planar waveform,” or “differentially orientated,” while those relating to cell populations include terms like “slightly elongated,” “plump,” “acellular areas,” and “regularly arranged” [12, 14, 16, 45, 81, 82, 83, 84, 85, 86]. This descriptive/qualitative approach allows for greater subjective interpretation, and limits capacity to standardize current knowledge and understanding of normal tendon structure, particularly differences between specific normal/healthy tendons. As an example, two investigations by the same group separately evaluated human Tibialis Anterior (TA) [85] and Tibialis Posterior (TP) [83] tendon structure. The descriptive “positive” Alcian‐Blue staining reported, gives no indication of spread or intensity, and may inadvertently suggest similarities when comparing these two tendons. In contrast, quantitative evaluation of the thickness of the fibrocartilaginous layer at the gliding zone of the respective malleoli presents clearly defined differences: TA = 30–50 μm [85] and TP = 100–200 μm [83].

## 5 Within‐Tendon Variations

A total of 22 publications assessed within‐tendon *Regional* variations (Table 4, Supporting Information S3: Table 2b). Mostly these studies explore variations across the longitudinal and transverse (cross‐sectional) planes of the tendon/ligament, although some evaluate differences between the IFM versus FM [55, 56, 66] or between individual bodies of tendons grouped together by a shared insertion point for example, subtendons forming the Quadriceps [38]. Only 4/22 studies explored regional variations within human tendons [27, 30, 40, 48]. No studies have reported a systematic investigation of regional variations across a range of normal tendons (human or animal) to determine whether regional patterns are consistent or different between tendons. Regional differences in a single anatomical tendon were evaluated in 15/22 papers (Supporting Information S3: Table 2b), and in those that evaluated more than one tendon‐type, regional differences were typically described independently. Only three studies directly compared *Region* and *Tendon*: Stanley et al. [36] studied equine superficial digital flexor tendons (SDFT) and common digital extensor tendons (CDET), however central versus core regions were independently assessed only in SDFT (Supporting Information S3: Tables 2a and 2b); Thorpe et al. [55] and Spiesz et al. [66] compared IFM and FM structure within equine SDFT and CDET (Supporting Information S3: Table 2b) and also between tendons (Supporting Information S3: Table 2a).

### 5.1 Transverse Differences

Cross‐sectional variations within tendons (e.g., central‐vs*.*‐peripheral, palmar‐vs*.*‐dorsal, medial‐vs*.*‐lateral, etc.) likely result in response to and modulate the tissue's capacity to function under variable loading (tensile or compressive) conditions. A variety of cross‐sectional differences have been reported (Supporting Information S3: Table 2b) including: greater posterior versus anterior fibril density in human Patellar tendons [40], longer crimp wavelengths in superficial versus deep zones of caprine infraspinatus tendons [33], and more ovoid cells in the central versus peripheral equine SDFT [36]. One of the most common cross‐sectional regional comparisons were central (core) versus peripheral in the equine SDFT (Supporting Information S3: Table 2b), with findings often contradictory, for example crimp angle and wavelength were variably greater or lesser in central regions [21, 22, 23, 26]. Factors, such as *Age* [22, 23] or *Lifestyle* [23, 36] have been suggested to play a role in these disparate *Regional* structural variations. Cross‐sectional differences may also vary with longitudinal location, for example, Williams et al. [37] found medial/lateral fibril diameters and fibril‐occupied areas varied between middle and distal rabbit Patellar tendon regions.

### 5.2 Longitudinal Differences

Proximal‐versus‐distal differences could play an important role in appendicular tendon function as they traverse a joint or in the transition of tendon to muscle and bone, reflecting changes in loading and biomechanical properties in these areas. Differences identified in the literature include significantly greater GAG content in the bone‐versus muscle‐end of ovine SDFT [57] and human long head of biceps tendon (LHBT) [48], and greater GAG chain length in distal (bone‐end) bovine SDFT [67] (Supporting Information S3: Table 2b). Mazzocca et al. [48] also found greater fibrillar organization in the distal (muscle‐end) compared to proximal (bone‐end) LHBT. This is consistent with more unorganized collagenous structures closest to the bone in the human Patellar tendon [27], suggesting commonality of less organized collagen at tendon‐bone junctions. Conversely, vascular density was reported to increase from proximal to distal in human TA tendons [30], while in ovine SDFT the middle portion had higher vascular density relative to proximal or distal regions [57]. Interestingly in canine Long Digital Extensor Tendon (LDET), SDFT, ACL, and Medial Collateral Ligament (MCL), Kharaz et al. [63] noted no longitudinal variation for any histological measurements. Collectively these and other studies [29, 67], suggest tendon‐ and species‐specific variability in longitudinal characteristics of normal tendons that require systematic exploration.

### 5.3 Hierarchy‐Related Differences

Three studies investigated differences between hierarchical tendon structures, specifically IFM and FM (Supporting Information S3: Table 2b). IFM had higher cell density in equine SDFT [56], while Spiesz et al. [66] found lower collagen content/organization (birefringence) in IFM in both equine CDET and SDFT with no tendon‐specific differences. Conversely, equine CDET and SDFT had tendon‐specific IFM and FM protein compositions [55]: CDET IFM had significantly higher lumican immuno‐staining than FM, while SDFT IFM had greater biglycan, lubricin, and elastin staining than FM. As discussed in a later section, the older age of the horses in these studies may contribute to tendon‐specific differences in IFM versus FM compositions [58].

### 5.4 Future Study Design Considerations

**Figure 2:**
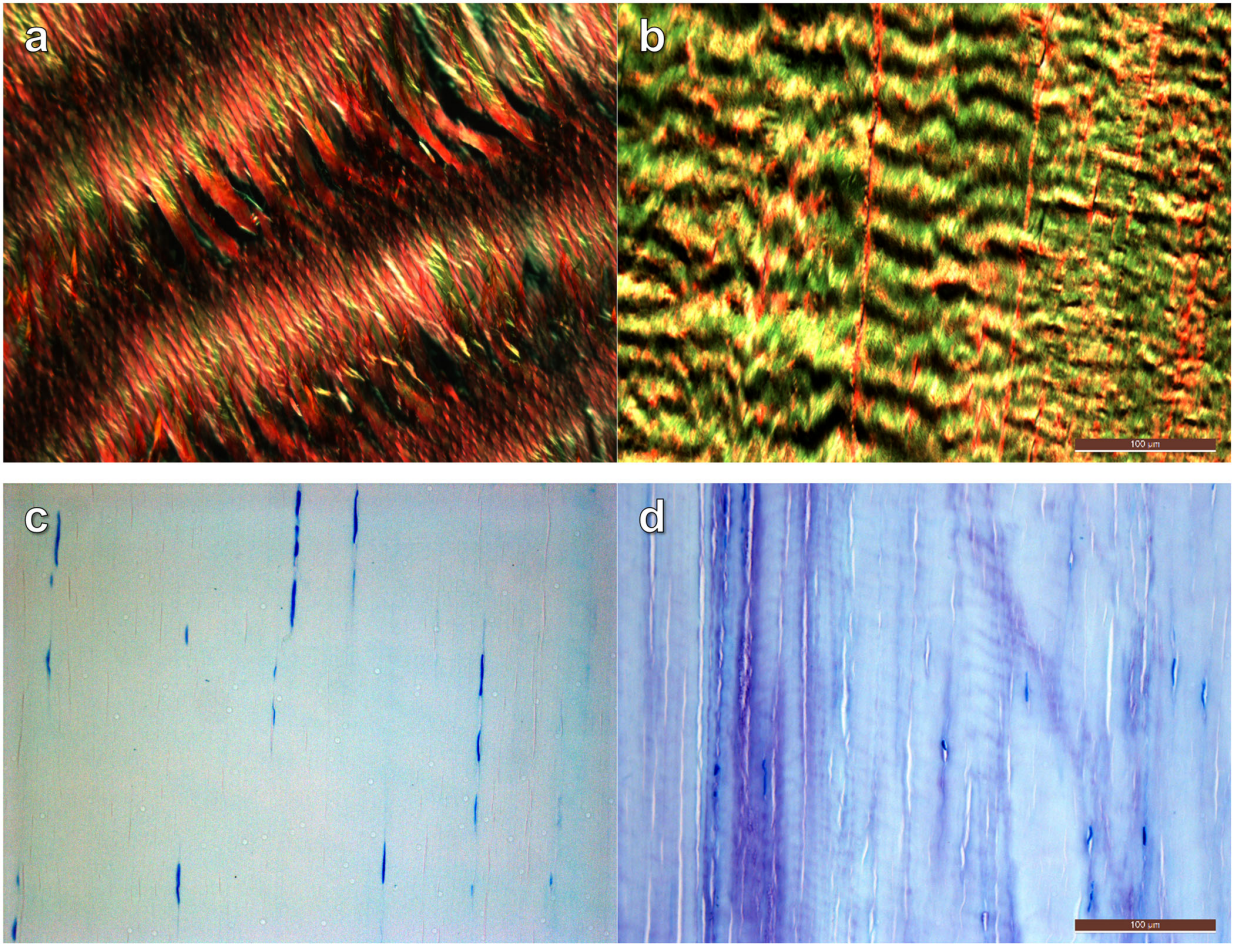
Light microscopy images showing variation in “normal” human tendons. Histological samples are from cadaveric lower‐leg tendons of female donors aged 71 (a and b) and 78 (c and d) years old. Polarized Picrosirius Red stained sections (a and b) depicting regional differences in crimp wavelength between matched proximal (a) and distal (b) regions of the Tibialis Anterior tendon. Toluidine Blue stained sections (c and d) depicting regional differences in proteoglycan content (purple staining) between matched proximal (c) and distal (d) regions of the Achilles tendon. Longitudinally orientated, formalin‐fixed, paraffin embedded histological sections at 4.5 µm thick. All images: ×200 magnification; scale bars = 100 µm (applies to all images of corresponding stains).

The aforementioned examples highlight that it is critical to explicitly state the region of sample collection and have region‐matched controls to evaluate pathological change. As an example, Cetti et al. [87] compared calcaneal‐insertional, rupture, and proximal (10 cm from calcaneus) regions in ruptured Achilles tendons but only a single control sample from the uninjured, contralateral tendon selected between 2.2 and 8.4 cm from the calcaneal insertion. The rupture site showed greater pathology (collagen degeneration, tenocyte necrosis, inflammation) than other regions in the injured tendon, and interestingly proximal‐tendon had greater pathology than distal‐tendon. However, as control samples were from various regions along the length of the uninjured tendon, no conclusions can be made about underlying regional differences that might alter injury susceptibility or changes postrupture. Our own preliminary analysis of normal/healthy human cadaveric tendons [88, 89], identified clear longitudinal regional differences in crimp wavelength (Figure 2a,b) and proteoglycan content (Figure 2c,d), reaffirming the importance of region‐matching control tissue samples.

In a series of studies in different animal models [17, 57, 90, 91, 92] we assessed topographical‐response to injury highlighting the importance of region‐matched controls. For example, Jacobson et al. [17] collected 12 proximal/distal and medial/lateral defined regional samples from each injured/pathological and contra‐lateral/uninjured SDFT in horses, identifying distinct regionally‐dependent histological differences. Cellularity, cell morphology, vascularity, cellular infiltration, collagen alignment, and proteoglycan content scores were significantly increased in injured versus control samples in the most proximal medial (*except collagen alignment score) and lateral (*except proteoglycan score) regions, while in the most distal regions, injury only increased cell morphology, vascularity and proteoglycan scores medially, and cell morphology and proteoglycan scores laterally. Detailed analysis regarding variations between control sample regions were not reported, except for highlighting the expected increase in proteoglycan content near bony‐attachment sites [17, 91] as identified in other studies [48, 57] (Supporting Information S3: Table 2b). A systematic study on normal (rather than pathological) regional histological characteristics in healthy tendons would increase understanding of how and why tendon response to injury or treatment differs.

## 6 Age‐Related Differences

We identified 30 publications which statistically assessed *Age* effects, with 12 exploring human tissue (Table 4, Supporting Information S3: Table 2c). It is important to note that these include studies evaluating histological changes in tendon occurring with increasing biological age both postnatally (i.e., during growth/maturation) and postmaturation (i.e., across the adult lifespan). It is recognized that the changes occurring in these different life phases may differ [93, 94], with such processes likely variable between tendons, species, or even the sexes. However, the inconsistency in postnatal times at which species and sexes reach maturity hinders comparison between different animal or human models and limits the ability to separately evaluate current literature across these different age/life‐stages. Additionally, the majority of studies which study *age‐*related changes utilize only two differently aged cohorts and compare between them rather than assessing continuous changes throughout the maturational/aging processes. Future work to better identify and define different life stages and understand specific event‐related structural homeostasis or associated changes is required.

The largest specified age span in the human studies was 66 years [51], while most spanned 29–36 years. In 6 of the 10 human studies which specified ages (two did not specify), donor average age was > 45 years, while one investigated child and adolescent cohorts [71]. In consideration of the preceding discussion of tendon variables, 5/12 human studies utilized only one anatomical tendon, and 4/12 only two. Two studies with > 2 tendons [32, 34] analyzed *Age*‐effects for only 2 of the study outcomes (collagen fibril thickness and density; no correlations found). Lastly, Johnson et al. [76] statistically assessed total histopathological score but not individual structural parameters across four rotator cuff tendons.

**Figure 3:**
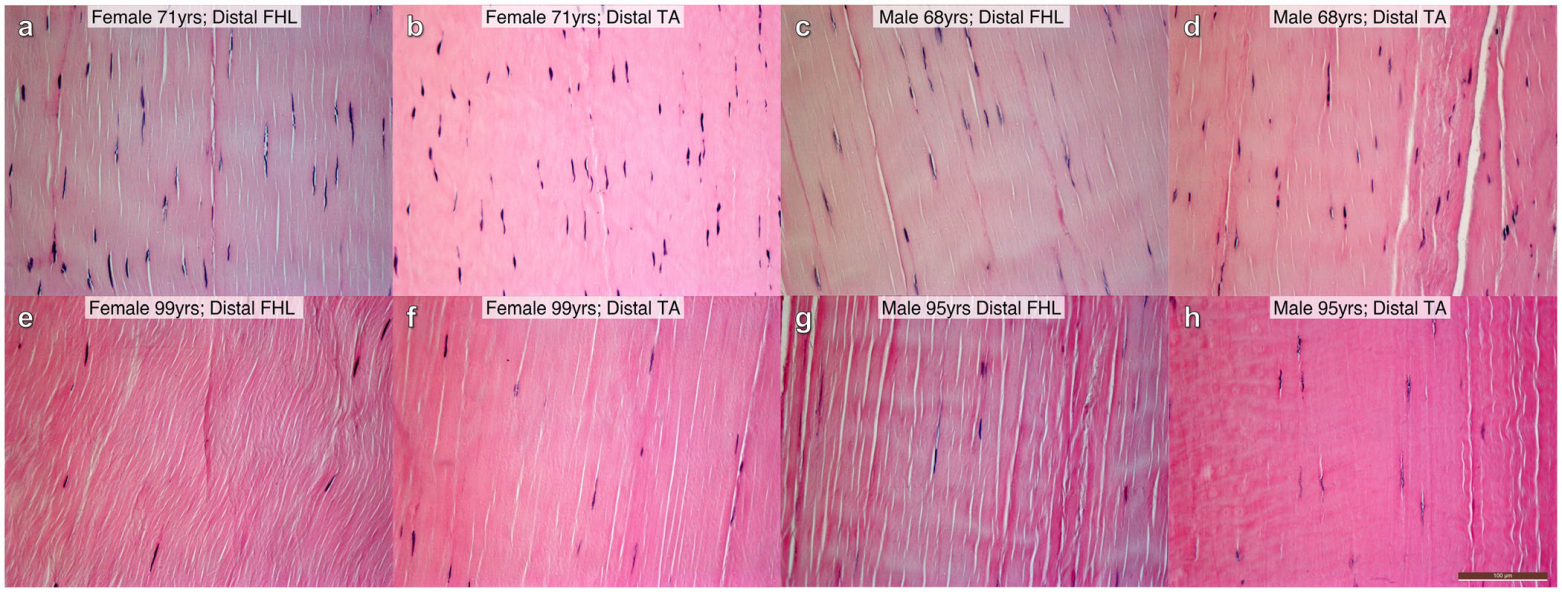
Light microscopy images showing cell density variation in “normal” human tendons. Histological samples are from the distal region of cadaveric lower‐leg Flexor Hallucis Longus (FHL; a, c, e, g) and Tibialis Anterior (TA; b, d, f, h) tendons. Female donors aged 71 (a and b) and 99 (e and f) years old, and male donors aged 68 (c and d) and 95 (g and h) years old. Note differences in relative cell density between younger (a–d) and older (e–h) donors. Longitudinally orientated, formalin‐fixed, paraffin embedded histological sections at 4.5 µm thick; Haematoxylin and Eosin staining. All images: ×200 magnification; scale bar = 100 µm (applies to all images).

*Age*‐associated changes have been reported for tendon cellular content/features, structural characteristics, and compositional makeup (Supporting Information S3: Table 2c). Reduced cellularity [46, 60, 68, 69, 71] and increased cellular rounding [60, 69] have been observed with increasing age in various animal tendons. We identified similar decreasing cell density with aging (Figure 3) in cadaveric human lower‐leg tendons from male and female donors aged 68–99 years [88, 89]. Importantly as discussed above, changes with biological age may not be linear, for example Legerlotz et al. [50] found cell density drastically decreased after 1 week of age in mice, but then gradually increased again from 6 weeks to 6 months of age likely reflecting changes associated with stages of maturation versus aging. Likewise, cell nuclear aspect ratios increased from 1 to 8 and 8 to 19 months in rats and then became more rounded between 19 and 28 months of age [77]. Furthermore, age‐related changes may differ between tendons for example, vascular density had a quadratic relationship across ages (18–68 years) in human Gluteus Medius tendons [59], while Prado et al. [30] reported no age:vascular‐density correlation in human TA tendons.

Many studies report no correlations/differences in fibril diameter with age [22, 24, 31, 32, 34, 73]. However, Shu et al. [65] reported both Achilles and tail tendons have significantly larger fibril diameters in skeletally mature (12 week‐old) compared to young (3 week‐old) mice. Similarly, Asai et al. [71] found “adult” (average 35 years) human Semitendinosus tendons had significantly larger fibril mean diameters compared to “immature” tendons (average 10 years), revealing changes in the finer collagenous organization between pre‐ and post‐maturation. Disparate crimp wavelength variations with age were reported in equine SDFT with shorter wavelength in older (average 11 years) compared to younger (average 2.75 years) [21] versus mouse tail tendon (shortest crimp in youngest 3 week‐old compared to 6 week‐, 9 week‐, 12 week‐, and 6 month‐old cohorts) [50].

While these studies reveal some *Age*‐associated cellular and structural differences, the heterogeneities reflect limitations in exploring these characteristics across different tendons and age brackets. Furthermore, direct comparison between studies is hampered by differing methodologies and potential species‐specific differences in chronological‐ versus biological‐age relationships. There is a major gap in knowledge of normal tendon aging throughout the human lifespan, particularly considering the narrow focus of specimens and outcome measures.

### 6.1 Age Versus Pathology

Histological research on aging of human tendons primarily focuses on injured/pathological samples with an unclear distinction between age‐related versus injury/disease‐driven changes. As an example of this poor division, the concept of “degeneration” is often used for both aging [95, 96, 97] and pathology [10, 98, 99] despite the potential for disparate mechanistic pathways in these two processes [100, 101]. Furthermore, older age groups generally exhibit more severe pathological change in diseased/injured tendons than younger ages for example, in rotator cuff tears [99, 102], Supraspinatus tendinitis [103], and Achilles tendinopathy [104]. While age was significantly correlated with histopathology‐score in a study of 155 Achilles tendon biopsies from individuals with symptomatic tendinopathy, age was also inversely correlated to lifestyle activity levels [104]. Differences in older populations could indicate “normal” physiological processes including those reflecting more sedentary lifestyles. It is important to consider differences between healthy or “normal” age‐related changes or whether aging causes pathology, notwithstanding such a division remains difficult to define.

### 6.2 Intersection of Age and Anatomical Tendon

While age evidently influences tendon properties, the interaction between age and different anatomical tendons requires consideration, as functionally distinct tendons can be affected differently. In horses for example, the energy‐storing SDFT contains significantly more elastin (predominantly in the IFM) than the positional CDET, but IFM elastin content and FM elastin organization decreased with age only in SDFTs, while FM elastin content increased significantly with age only in CDETs [58]. Age also led to the accumulation of partially degraded collagen [98] as well as increased IFM‐stiffness [49] in SDFTs and not CDFTs (potentially explaining the age‐related prevalence of injury in the SDFT). Unfortunately, less than half of the studies exploring *Age*‐related differences (13/30; Supporting Information S3: Table 2c) assessed more than one tissue/tendon type. It is important for future histological studies on different tendons to consider age as a factor, particularly when existing knowledge for those specific tendons, species and ages is absent.

### 6.3 Study Design and Methodology Considerations

The typically older age of human cadavers is a limiting factor for investigations utilizing “normal” controls or investigating normal tendon structure. When control specimens are taken from deceased patients [16, 105, 106, 107, 108, 109, 110, 111], it is not uncommon for the age difference from the often younger, more active and injury‐prone cohort‐of‐interest to be in the order of 20 years [105, 106, 107, 110, 111]. For example, a histological study of quadriceps tendon ruptures compared biopsies from patients aged 28–81 years (mean 51.3) to cadaveric controls aged 66–91 years (mean 74.5) [107]. Comparative patient biopsies may also be from different age cohorts due to circumstances/opportunities for tissue collection for example, Zhu et al. [45] compared normal hamstring tendon grafts from patients undergoing surgery for ACL rupture (average age 25.3 ± 7.3 years) to “normal” ACL tissue from osteoarthritis patients undergoing total knee arthroplasty (average age 65.3 ± 5.6 years). It is critical that future studies better characterize “normal” tendon across a wide age‐range which will help overcome issues of age‐matching control and experimental samples.

Another confounding factor for *age*‐effects is the potential for *species*‐related differences. Conflicting results have been reported for collagen fibril diameter, which was not correlated with age in many (but not all) human studies [31, 34, 73], but was affected in equine [28], rat [20], and mouse [65] tendons. Whether this reflects bona fide species‐specific differences, the particular tendons evaluated, other environmental influences (e.g., activity [20, 28]), or older chronological age in human studies is unclear. Study methodology may also affect outcomes as shown by Ingelmark [20] who used electron microscopy of fresh versus osmium tetroxide (OsO_4_) impregnated samples to assess fibril diameters in old and young rats in response to different activity levels. OsO_4_ treatment significantly reduced fibril diameters compared to fresh samples, however fresh samples showed no difference in fibril diameters between groups, while OsO_4_ showed larger diameters for the old trained versus young untrained rats [20]. The complex age‐ and exercise‐related differences in response to OsO_4_ undoubtedly reflect changes in tendon biology but may not actually be indicative of altered fibril size in vivo. Likewise, tissue orientation affected significant findings of histological vascular density in the human Gluteus Medius tendon where correlations between density and age were observed in longitudinal but not transverse sections [59].

## 7 Sex‐Related Differences

A noticeable gap in the literature is understanding of *sex*‐related effects on tendon structure. Only 8 papers explored *Sex* as a variable microscopically, with over half (5/8) investigating human tendons (Table 4, Supporting Information S3: Table 2d). Of the eight studies only one found a significant correlation between sex and structural characteristics: greater cellularity in the Achilles tendons of female compared to male mice [61]. No other studies reported sex‐differences in cell density or morphology in various tendon/ligament models [54, 69, 71]. Pardes et al. [54] also found no differences between male, female or ovariectomized‐female rat Achilles tendon proteoglycan staining. No correlations were found between sex and thickness or density of collagen fibrils in tendons commonly used for human grafts [31, 32, 34], and Prado et al. [30] identified no sex‐related differences in the vascular densities of human TP tendon.

The scope of research available on *Sex*‐related differences in microscopic tendon structural properties is limited, with the major focus being on cellular features and very few studies reporting on these or other structural characteristics (Supporting Information S3: Table 2d). It is common for studies investigating animal tendons to omit reporting sex or to use mixed (pooled) populations [14, 22, 55, 63, 112, 113, 114], often because it is unknown to the researcher (e.g., tendons from discarded limbs at an abattoir). Surprisingly, these issues are also observed in many human studies, where sex is either not disclosed [62, 99, 109, 115, 116] (potentially as a result of ethical/privacy issues), or *sex* as a variable is not included in the analyses due to limited sample availability [10, 27]. While the few studies available suggest limited differences between sexes, it is important to continue to fill this gap to fully understand the effects that patient demographics have on tendon structure. From our own study exploring a range of histological outcomes in nine different cadaveric human lower‐leg tendons (donors aged 68–99 years; five males, four females), females had significantly longer crimp wavelengths relative to males (manuscript in preparation). These types of structural differences may be implicated in reported sexually dimorphic tendon mechanical attributes [23, 117, 118, 119], which could influence the intervention approach or outcomes of tendon related injury. Large‐scale studies exploring tendon variations and demographic differences including *Sex*‐specific analyses are needed.

## 8 Other Normal/Healthy Tendon Variabilities

In addition to the primary “variables of interest,” 16 papers assessed *Other* factors such as species or lifestyle differences (e.g., exercise, diet, etc.) on tendon structure (Table 4, Supporting Information S3: Table 2e).

### 8.1 Species

Of the initial 105 eligible studies (Supporting Information S2: Table 1), only four incorporated more than one species, with three not conducting statistical *Species* comparisons. Aune et al. [120] used a rat model and human biopsies to study nerve regeneration following ACL reconstruction with Patellar tendon autografts but only observational between‐species comparisons were made. Likewise, Tinguely et al. [77] reported only graphical data comparing rat and human aging and injury responses in tendons with no direct statistical comparisons. Sato et al. [121] compared the tendon healing of mice to that of Iberian ribbed newts, however control/normal tendons were not statistically compared. Gsell et al. [75] was the only paper specifically comparing tendons from different *Species*, exploring collagen fibril properties from bovine and rat tendons. However, the primary purpose of the study was investigating differences between positional (bovine LDET and rat Tail tendon) and energy storing (bovine SDFT) tendons. While differences were observed between the three tendon‐types, species‐specific comparisons were not a focus and use of different anatomical tendons limits interrogation of species‐specific influences on functionally “like‐for‐like” tendons.

### 8.2 Other Comparisons

*Other* variables' effects on normal/healthy tendon structural characteristics were explored in 15 papers (Supporting Information S3: Table 2e), with 6 of these studies having this as the only “variable of interest” assessed. Variables explored included: *Lifestyle* differences (e.g., level of physical activity/training/exercise [20, 27, 28, 36, 42, 52, 64], or diet [72]); *Comorbidities*/general health status (e.g., diabetic vs*.* nondiabetic conditions [25] or unrelated musculoskeletal injuries [27]); *Demographic* information (e.g., weight [27, 69, 71], body mass index [71], and height [27, 71]); or *Other* correlations (e.g., hormonal status [39, 54], left vs*.* right limbs [30], or even specifics of individual subjects [33]). Unsurprisingly considering the load bearing role of tendons, exercise‐related (*Lifestyle*) comparisons predominate the *Other* variables that significantly modulated tendon histological structural measures. For example, increased training/exercise was found to be related to a reduction in the proportion of spindle‐shaped tenocyte nuclei [36], and an increase in collagen alignment [42]. However, opposing results for fibril diameter changes following training (studies reporting an increase in diameter [20] compared to a decrease in diameter [28]), and the response of differing training conditions (strength, resistance, hypertrophy, or no training) causing different effects on collagenous structure (birefringence properties) [64] highlight the multifactorial influences which may predict structural variations.

## 9 Conclusions

It is self‐evident that knowledge of normal variations in tendon histo‐anatomical structure is essential for conducting studies on tendon structure‐function relationships, clinical pathology, and response to treatment; particularly when current practice in clinical and research settings utilizes histopathological scoring systems which grade characteristics relative to a normal/control reference [27, 122, 123]. To date, no studies have systematically investigated structural variations between a range of different normal tendons using consistent methodologies. This is a major limiting factor in current tendon research and is critical foundational knowledge for future studies.

Tendons have a complex and varied structure, resulting in many considerations when it comes to understanding the structure‐function relationships of the tissue. Although a considerable amount of literature is available on tendon structural/compositional variations, this knowledge is predominantly derived from studies which: (1) focus on pathological conditions/structural changes; (2) report only descriptive classifications; (3) pertain to only one or two distinct tendons and offer limited comparability to other investigations; or (4) assess limited outcomes, for example, only biochemical data with no other studies performed on the tissues for more comparative analysis. Accordingly, current knowledge of tendon structural characteristics and normal variations in response to variables such as the specific anatomical *Tendon*, *Region*, *Age*, and *Sex* is limited. As a result, other areas of tendon research such as investigations into tendon physiological and pathophysiological mechanisms, biomechanical properties, or clinical outcomes, are inhibited by a lack of understanding of structural “baselines” and natural normal/healthy tissue variations for different tendons.

A structure must be known before a structure‐function relationship can be fully understood. Similarly, normal must be known before an appreciation of abnormal can be achieved. This review has highlighted that further research is needed to quantitatively characterize normal tendon structure in greater detail and identify variations across a range of different tendons with consideration of influential variables. Box 1 identifies the key gaps in current knowledge of tendon structural variations that need to be addressed in future studies.

Box 1 **Summary of knowledge gaps in the histological analysis of tendons.**

Currently, there are limited studies which compare the multi‐scale structural variations of normal (healthy) tendons. Further structural (e.g., histological, microscopy) investigations are needed to:

1. Systematically compare variations between a wide range of different anatomical tendons,
2. Characterize variations within tendons, for example, within the longitudinal or cross‐sectional planes, and to determine whether any such variations exist in a range of different tendons,
3. Understand the effects of age on tendon structure, and the interaction between “normal” aging and sedentary lifestyle versus injury, pathology, or disease.
4. Compare the above outcomes between the sexes,
5. Appreciate species‐specific variations as well as other variables that may influence tendon structure, for example, lifestyle, demographics, comorbidities, and so forth.
6. Understand how confounding factors might result in differences for example, specific tendons exhibiting different regional, age or other variations.
7. Appreciate how differing methodologies may influence research findings and translatability across the field.

Characteristics that have been identified as important and widely explored features of tendons, that should be considered for assessment in future tendon research include, but are not limited to:

i. Cell populations (e.g., number/density, morphology etc.)
ii. Compositional make‐up (e.g., location, amount, spread etc. of different components)
iii. Overall structural architecture (e.g., fibril/fiber/fascicle organization, collagen alignment, crimp patterns etc.)

## Author Contributions

The first author conducted the literature search described in this review. All authors contributed to the writing and preparation of this manuscript. All authors have read and approved the final submitted manuscript.

## Ethics Statement

The histological images depicted in Figures 2 and 3 were obtained as part of a project approved by Northern Sydney Local Health District Human Research Ethics Committee (NSLHD HREC, Sydney, Australia) in accordance with the National Health and Medical Research Council (NHMRC) Act 1992. Human ethics approval code: 2019/ETH08332.

## Conflicts of Interest

The authors declare no conflicts of interest.

## Supporting information

Supporting information.

Supporting information.

Supporting information.

## Data Availability

Additional Supporting Information may be found in the online version of this article.
