# Supplementary material for: Structural Variations of Tendons: A Systematic Search and Narrative Review of Histological Differences Between Tendons, Tendon Regions, Sex, and Age
Source: J Orthop Res. 2025 Feb 26;43(5):994–1011. doi: 10.1002/jor.26060 (PMC11982604; doi:10.1002/jor.26060)
Supplement: Supplementary file 1 — Supporting information. [file JOR-43-994-s001.docx]

# Supplementary Material A – Systematic Search Terms

To explore variations in normal/healthy tendon multiscale structure, an electronic database search of PubMed (MEDLINE) was initially conducted on 30^th^ September 2022 using the keyword search terms as follows: (tendon OR tendons OR tend*) AND (histolog* OR histochem* OR microscop*) AND (normal OR healthy OR "non-pathological" OR "non pathological") AND (structu*) AND (compar* OR differen* OR variation OR variations). During the course of reviewing the literature, the search was conducted again on 22^nd^ September 2023 and 17^th^ August 2024 to capture new records. To ensure results remained consistent, the full list of search and MeSH terms as per the initial keyword search in 2022 were used for subsequent searches to ensure all articles captured reflect the results generated from the indexed National Library of Medicine (NLM) terms list at the time of the initial search. The full list of terms used were: ("tendinopathy"[MeSH Terms] OR "tendinopathy"[All Fields] OR "tendonitis"[All Fields] OR "tendon s"[All Fields] OR "tendonous"[All Fields] OR "tendons"[MeSH Terms] OR "tendons"[All Fields] OR "tendon"[All Fields] OR ("tendinopathy"[MeSH Terms] OR "tendinopathy"[All Fields] OR "tendonitis"[All Fields] OR "tendon s"[All Fields] OR "tendonous"[All Fields] OR "tendons"[MeSH Terms] OR "tendons"[All Fields] OR "tendon"[All Fields]) OR "tend*"[All Fields]) AND ("histolog*"[All Fields] OR "histochem*"[All Fields] OR "microscop*"[All Fields]) AND ("normalisation"[All Fields] OR "normalisations"[All Fields] OR "normalise"[All Fields] OR "normalised"[All Fields] OR "normalises"[All Fields] OR "normalising"[All Fields] OR "normalization"[All Fields] OR "normalizations"[All Fields] OR "normalize"[All Fields] OR "normalized"[All Fields] OR "normalizer"[All Fields] OR "normalizers"[All Fields] OR "normalizes"[All Fields] OR "normalizing"[All Fields] OR "normally"[All Fields] OR "normals"[All Fields] OR "tissues"[MeSH Terms] OR "tissues"[All Fields] OR "normal"[All Fields] OR ("healthies"[All Fields] OR "healthy"[All Fields]) OR "non-pathological"[All Fields] OR "non-pathological"[All Fields]) AND "structu*"[All Fields] AND ("compar*"[All Fields] OR "differen*"[All Fields] OR ("variation"[All Fields] OR "variations"[All Fields]) OR ("variation"[All Fields] OR "variations"[All Fields])).
