# Supplementary material for: Structural Variations of Tendons: A Systematic Search and Narrative Review of Histological Differences Between Tendons, Tendon Regions, Sex, and Age
Source: J Orthop Res. 2025 Feb 26;43(5):994–1011. doi: 10.1002/jor.26060 (PMC11982604; doi:10.1002/jor.26060)
Supplement: Supplementary file 3 — Supporting information. [file JOR-43-994-s003.docx]

**Table S-2a.** Primary findings relating to differences between tendons/ligaments. Tendons/Ligaments or Outcomes of the study not relevant or statistically assessed for the detailed variable (*Tendon*) are greyed-out. Bold **outcomes** indicate those with statistically significant findings. *Refer to Table 5in manuscript for acronym definitions.*

| Reference | Species | Tendon (T) / Ligaments (L) | Outcomes | Major (statistically significant) findings |
| --- | --- | --- | --- | --- |
| Birch et al. (1999) | Equine | **2T**  DDFT, SDFT | **Fibril diameter** | DDFT had larger fibril diameter than SDFT. |
| Hadjicostas et al. (2007a) | **Human** | **2T**  Patellar, Quadriceps | **Collagen-fibril density**, Fibril diameter, Vascular density, **Cell density** | Quadriceps had higher fibril density (fibril:interstitium ratio) than Patellar tendon.  Quadriceps had higher cell density than Patellar tendon. |
| Hadjicostas et al. (2007b) | **Human** | **3T**  Gracilis, Semitendinosus, Patellar | **Collagen-fibril density**, Fibril diameter, Vascular density, **Cell density** | Both Gracilis and Semitendinosus had higher fibril density (fibril:interstitium ratio) than Patellar tendon.  Both Gracilis and Semitendinosus had higher cell density than Patellar tendon. |
| Hadjicostas et al. (2008a) | **Human** | **4T**  Gracilis, Semitendinosus, Patellar, Quadriceps  **1L**  ACL | **Collagen-fibril density**, Fibril diameter, **Vascular density**, **Cell density**, **Elastic fibril density**, **Collagen fibril-type density (Type I, Type III, Type V)** | Gracilis had higher fibril density (fibril:interstitium ratio) than Quadriceps / Semitendinosus / ACL / Patellar. Quadriceps / Semitendinosus had higher fibril density than ACL and Patellar tendon.  ACL had higher vascular density than Gracilis / Semitendinosus / Quadriceps / Patellar tendons. Semitendinosus had higher vascular density than Gracilis / Quadriceps / Patellar tendons. Quadriceps had higher vascular density than Patellar tendon.  ACL had higher cell density than Gracilis / Semitendinosus / Quadriceps / Patellar tendons. Gracilis / Semitendinosus / Quadriceps had higher cell density than Patellar tendon.  ACL had higher elastic fibril density than Gracilis / Semitendinosus / Quadriceps / Patellar tendons.  ACL had lower Collagen-I fibril density than Gracilis / Semitendinosus / Quadriceps / Patellar tendons. Gracilis had lower Collagen-I fibril density than Semitendinosus / Quadriceps / Patellar tendons.  ACL had higher Collagen-III fibril density than Gracilis / Semitendinosus / Quadriceps / Patellar tendons. Gracilis / Semitendinosus / Patellar had higher Collagen-III fibril density than Quadriceps tendon.  ACL had higher Collagen-V fibril density than Gracilis / Semitendinosus / Quadriceps / Patellar tendons. Gracilis had higher Collagen-V fibril density than Semitendinosus / Quadriceps tendons. |
| Hadjicostas et al. (2008b) | **Human** | **2T**  Gracilis, Semitendinosus | **Collagen-fibril density**, Fibril diameter, **Vascular density**, Cell density, Elastic fibril density, Collagen fibril-type density (**Type I**, Type III, **Type V**) | Gracilis had higher fibril density (fibril:interstitium ratio) than Semitendinosus.  Semitendinosus had higher vascular density than Gracilis.  Gracilis had lower Collagen-I fibril density than Semitendinosus.  Gracilis had higher Collagen-V fibril density than Semitendinosus. |
| Stanley et al. (2008) | Equine | **2T**  CDET, SDFT | **Cell density**, **Cell morphology** | Consistently across all three different study (exercise) models:  CDET had lower total cell density than SDFTc (central/core region) and SDFTp (peripheral region).  CDET had lower type 1 (spindle) cell density than SDFTc and SDFTp.  For Long-term exercise and Foal studies:  CDET had lower type 2 (ovoid) cell density than SDFTc and SDFTp.  For the Foal study:  CDET had higher proportion of type 1 (spindle) cells than SDFTc and SDFTp.  For short-term exercise study:  CDET had lower type 2 (ovoid) cell density than SDFTc.  Additionally, CDET had lower proportion of type 1 (spindle) cells than SDFTc and SDFTp. |
| Franchi et al. (2009) | Murine (Rat) | **3T**  Patellar, RFT, VIT | **Crimp number**, Crimp angle, Crimp wavelength | VIT had a greater quantity of crimps than RFT and Patellar.  VIT had smaller crimp top angle than RFT and Patellar.  VIT had smaller crimp base length (wavelength) than RFT and Patellar. |
| Hosaka et al. (2010) | Equine | **3T**  CDET, DDFT, SDFT | **Cell density** | SDFT had greater cell density than DDFT and CDET. DDFT had greater cell density than CDET. |
| Weiss et al. (2012) | **Human** | **5T**  Gracilis, Semitendinosus, Patellar, Quadriceps, Tibialis Anterior  **1L**  ACL | Cell density*,* ***αSMA+ cell density****,* ***Crimp wavelength*** | There was a significant correlation of myofibroblast density and crimp frequency for ACL, Semitendinosus, Gracilis, Tibialis Anterior, Quadriceps, and Patellar tendon. Correlation strength was greatest for Patellar, followed by Semitendinosus, ACL, Quadriceps, Tibialis Anterior, and lastly Gracilis tendon. |
| Zhu et al. (2012) | **Human** | ***1T**  "Hamstring" (Gracilis or Semitendinosus)  **1L**  ACL | **Fibril diameter**, **Fibril density** | ACL had greater percentage of 0-50nm and 50-100nm fibrils than Hamstring tendon. Hamstrings had greater percentage of 100-150nm and 150-200nm fibrils than ACL.  ACL had higher mean density of fibrils than Hamstrings. |
| Tilley et al. (2014) | **Human** | **2T**  Subscapularis, Supraspinatus | **Fibril diameter**, Fibril angle (orientation), Crimp wavelength, **Fibre-thickness ratios (PSR staining)** | Supraspinatus tendon (young cohort) had thinner fibril diameters than Subscapularis tendon (old cohort).  Supraspinatus tendon (young cohort) had lower ratio of thick-to-thin diameter fibrils than Subscapularis tendon (old cohort). |
| Herod et al. (2016) | Bovine | **2T**  CDET, SDFT | **Crimp wavelength**, **Fibril diameter** | CDET had longer crimp wavelength than SDFT.  CDET had larger (diameter) collagen fibrils than SDFT. |
| Thorpe et al. (2016a) | Equine | **2T**  CDET, SDFT | Elastin content, GAG content (IHC staining intensity: Biglycan, Decorin, **Fibromodulin**, Lubricin, **Lumican**) | Within the IFM, CDET had greater lumican staining than SDFT.  Within the FM, CDET had greater fibromodulin staining than SDFT. |
| Godinho et al. (2017) | Equine | **2T**  CDET, SDFT | **Elastin content** (IHC staining), **IFM:FM area ratio**, **Elastin fibre orientation/alignment** | Within the younger cohort, SDFT had higher IFM elastin content than CDET.  Within both cohorts, SDFT had higher FM elastin content than CDET.  Within both cohorts, SDFT had higher IFM:FM area than CDET.  Within the older cohort, SDFT had more randomly distributed elastin fibres than CDET.  Additionally, within the older cohort, SDFT had less overall organisation than CDET. In both cohorts, SDFT had less elastin aligned parallel to the IFM and more elastin aligned perpendicular to the IFM than CDET. |
| Kharaz et al. (2018) | Canine | **2T**  LDET, SDFT  **2L**  ACL, MCL | **Microfibril (elastin) staining** (distribution, location and pericellular association), GAG staining (distribution, location, and pericellular association), Chondroid cell change, **Protein/GAG content,** **IHC staining** (Collagen type-I and -III, **Aggrecan**, **Versican**, Decorin, Biglycan, Elastin, Fibrillin-1, Fibrillin-2), **Collagen content (hydroxyproline)**, **Tissue architecture**, **Cell morphology**, Cell distribution, **Cell arrangement**, Vascularity, Inflammatory cell infiltration | The ACL had lower ECM organization than LDET and SDFT. MCL had lower ECM organization than LDET.  ACL had more rounded cell morphology and less aligned cells than LDET.  SDFT had less total collagen content than ACL, MCL, and LDET.  ACL had higher GAG content than MCL, LDET, and SDFT.  ACL had higher elastin content than MCL, LDET, and SDFT.  ACL had higher aggrecan staining and versican staining than MCL, LDET. |
| Spiesz et al. (2018) | Equine | **2T**  CDET, SDFT | ***Collagen fibre organisation (birefringence),*** **Crimp angle**, **Crimp wavelength** | In stained sections, CDET had higher total birefringence (higher collagen content / organisation) than SDFT.  CDET had larger crimp angle than SDFT.  CDET had longer crimp wavelength than SDFT. |
| Hayashi et al. (2019) | Canine | **1T**  LDET  **3L**  CaCL, CrCL, MCL | **Cell morphology (fusiform density, ovoid density, spheroid density)**, Cell density | MCL and LDET had greater densities of fusiform nuclei than in CaCL and CrCL.  CaCL and CrCL had greater densities of ovoid and spheroid nuclei than in MCL and LDET.  For Fusiform cell density, significant differences were observed between structures (not specified: CaCL < CrCL < MCL < LDET)  For Ovoid cell density, significant differences were observed between structures (not specified: CaCL > CrCL > LDET > MCL)  For Spheroid cell density, significant differences were observed between structures (not specified: CrCL > CaCL > LDET > MCL) |
| Ristaniemi et al. (2021) | Bovine | **1T**  Patellar  **4L**  ACL, LCL, MCL, PCL | **Crimp angle**, Crimp wavelength | Bovine ACL had smaller crimp angle than the MCL, PCL, and Patellar Tendon. |
| Fjordbakk and Marques-Smith (2023) | Equine | **3T**  Medial Patellar, Intermediate Patellar, Lateral Patellar | **IFM thickness**, IFM vascularity, Adipose Infiltration | Medial PT had thicker IFM than Intermediate PT and Lateral PT.  Intermediate PT had thicker IFM than Lateral PT. |
| Gsell et al. (2023) | **Bovine, Murine (Rat)** | **3T**  LDET [bovine], SDFT [bovine], Tail [rat] | **Fibril CSA**, **Fibril Aspect Ratio** | Bovine SDFT fibrils had smaller CSA than bovine LDET and rat tail fibrils.  Bovine LDET fibrils had smaller Aspect Ratio (aka flatter / less plump) than bovine SDFT and rat tail fibrils. |

**Table S-2b.** Primary findings relating to differences between regions within a tendon. Tendons/Ligaments or Outcomes of the study not relevant or statistically assessed for the detailed variable (*Region*) are greyed-out. Bold **outcomes** indicate those with statistically significant findings. *Refer to Table 5 in manuscript for acronym definitions.*

| Reference | Species | Tendon (T) / Ligaments (L) | Outcomes | Major (statistically significant) findings | Regions compared |
| --- | --- | --- | --- | --- | --- |
| Wilmink et al. (1992) | Equine | **1T**  SDFT | **Crimp wavelength**, **Crimp angle** | In the old cohort, Central fibres had smaller crimp angle than Peripheral fibres.  In the old cohort, Central fibres had shorter crimp wavelength than Peripheral fibres. | Central vs Peripheral |
| Patterson-Kane et al. (1997a) | Equine | **1T**  SDFT | Fibril diameter, **Crimp wavelength**, **Crimp angle** | In the young cohort, Central fibres had larger crimp angle than Peripheral fibres. In the old cohort, Central fibres had smaller crimp angle than Peripheral fibres.  In the young cohort, Central fibres had longer crimp wavelength than Peripheral fibres. | Central vs Peripheral |
| Patterson-Kane et al. (1997b) | Equine | **1T**  SDFT | **Crimp wavelength**, **Crimp angle** | For exercised equines (majority <10 years old), 6/9 horses found Central fibres had smaller crimp angle than Peripheral fibres. For non-exercised equines, 2/9 horsed found Central fibres had smaller crimp angle than Peripheral fibres, whilst 3/9 horses found Central fibres had larger crimp angle than Peripheral fibres.  For exercised equines (majority <10 years old), 3/9 horses found Central fibres had shorter crimp wavelength than Peripheral fibres, whilst 3/9 horsed found Central fibres had longer crimp wavelength than Peripheral fibres. For non-exercised equines, 2/9 horses found Central fibres had shorter crimp wavelength than Peripheral fibres, whilst 3/9 horsed found Central fibres had longer crimp wavelength than Peripheral fibres. | Central vs Peripheral |
| Birch et al. (1999) | Equine | **2T**  DDFT, SDFT | Fibril diameter |  | Central vs Peripheral |
| Gibson et al. (2002) | Equine | **1T**  SDFT | * Crimp wavelength, Crimp angle |  | Central vs Peripheral  (Proximal, middle, distal regions also included but not compared) |
| Cook et al. (2004) | **Human** | **1T**  Patellar | **Cell characteristics**, **Ground substance staining**, **Collagenous structure**, Vascularity | Proximal region tendon had “worse” cell characteristics (morphological) than the Distal region.  Proximal region tendon had “worse” ground substance staining than the Distal region.  Proximal region tendon had “worse” collagen structure than the Distal region. | Longitudinal:  Proximally (bone-attachment) vs 1cm distally |
| Watanabe et al. (2005) | Equine | **1T**  SDFT | **Fibril diameter (mean,** MAD**)**, **Collagen:non-collagen ratio** | Proximal region had smaller fibril diameters than Middle region and Distal region tendon.  Proximal region had greater collagen density (collagen:non-collagen ratio) than Distal region tendon. | Longitudinal:  Proximal Middle, Distal |
| Prado et al. (2006) | **Human** | **1T**  Tibialis Posterior | **Vascular density** | Longitudinal level (region) correlated with vascular density; Proximal tendon had lower vascular density than distal tendon. | Longitudinal:  6 segments |
| Funakoshi et al. (2008) | Caprine | **2T**  Infraspinatus, Patellar | Fascicle diameter, **Crimp wavelength**, | Superior aspect had longer crimp wavelength than the Deep (humeral side) aspect. | Transverse (Infraspinatus): Superior / superficial vs Deep) humeral joint side compared with the superior aspect of the tendon |
| Stanley et al. (2008) | Equine | **2T**  CDET, SDFT | Cell density, **Cell morphology** | Across all three exercise study models:  SDFT Central region had higher type 2 (ovoid) cell density than SDFT Peripheral region.  In short-term exercise and foal studies:  SDFT Central region had lower type 1 (spindle) cell density than SDFT Peripheral region.  Additionally, SDFT Central region had lower proportion of type 1 (spindle) cells than the SDFT Peripheral region.  In the Foal study:  SDFT Central region had lower type 1 (spindle) cell density than SDFT Peripheral region. | Central vs Peripheral |
| Williams et al. (2008) | Leporine (Rabbit) | **1T**  Patellar | **Fibril diameter**, **Fibril-occupied area**, **Fibril density** | Significant difference was found between all regions for fibril diameter: Central-Centre > Central-Distal > Medial-Centre > Central-Proximal > Lateral-Distal.  Lateral-Distal tendon had higher fibril density (number density) than Central-Distal, Central-Centre, Medial-Centre and Central-Proximal and tendon. Medial-Centre tendon had [slightly] lower fibril density than Central-Proximal tendon.  Central-Centre, Central-Distal and Lateral-Distal tendon had higher fibril-occupied area (Area Fraction) than Medial-Centre and Central-Proximal tendon. | Transverse and Longitudinal:  Central-Proximal (CP), Central-Centre (CC), Medial-Centre (MC), Central-Distal (CD), and Lateral-Distal (LD) |
| Franchi et al. (2009) | Murine (Rat) | **3T**  Patellar, RFT, VIT | **Crimp number, Crimp angle, Crimp wavelength** | VIT had greater crimp number than RFT.  VIT had smaller crimp angle than RFT.  VIT had shorter crimp wavelength than RFT. | Sub-tendon bodies (Quadriceps) |
| Hansen et al. (2010) | **Human** | **1T**  Patellar | Volume fraction of fibrils, **Fibril density**, Fibril diameter | Deep (posterior) tendon had higher fibril density than Superficial (anterior) tendon. | Transverse: superficial/anterior vs deep/posterior |
| Mazzocca et al. (2013) | **Human** | **1T**  LHBT | Protein content (IHC staining: Collagen type I, Collagen type III, Tenascin C, Decorin), **Proteoglycan content (Alcian blue staining)**, **Collagen fibre orientation/organisation** | Proximal (bone-end) tendon had higher proteoglycan content than distal tendon.  Proximal (bone-end) tendon had lower collagen organisation than distal tendon. | Longitudinal: Proximal vs Distal |
| Thorpe et al. (2016a) | Equine | **2T**  CDET, SDFT | **Elastin content**, GAG content (IHC staining intensity: **Biglycan**, Decorin, Fibromodulin, **Lubricin**, **Lumican**) | For CDET, IFM had greater lumican staining than FM.  For SDFT, IFM had greater biglycan staining than FM.  For SDFT, IFM had greater lubricin staining than FM.  For SDFT, IFM had greater elastin staining than FM. | IFM vs FM |
| Thorpe et al. (2016b) | Equine | **1T**  SDFT | **Cell density** | IFM had higher cell density than FM. | IFM vs FM |
| Biasutti et al. (2017) | Ovine | **1T**  SDFT | **Cellularity**, **Cell morphology**, **Vascularity**, **Inflammatory cell infiltration**, Collagen fibre alignment, **Proteoglycan content (TB staining)** | Significant (correlative) difference was found between tendon regions and cellularity (cell density): Middle tendon had the highest cell density than Proximal/Distal sites.  Significant (correlative) difference was found between tendon regions and cell morphology: Middle tendon had the highest (more rounded) cell morphology than Proximal/Distal sites.  Significant (correlative) difference was found between tendon regions and vascularity: Middle tendon had the highest vascular density than Proximal/Distal sites.  Significant (correlative) difference was found between tendon regions and inflammatory cell infiltration: Middle tendon had the highest infiltration than Proximal/Distal sites.  Significant (correlative) difference was found between tendon regions and proteoglycan content: Middle tendon had the highest proteoglycan content than Proximal/Distal sites. Distal tendon had higher proteoglycan content than the Proximal site. | Longitudinal: Proximal, Middle, Distal |
| Kharaz et al. (2018) | Canine | **2T**  LDET, SDFT  **2L**  ACL, MCL | Microfibril (elastin) staining (distribution, location and pericellular association), GAG staining (distribution, location, and pericellular association), Chondroid cell change, Protein/GAG content, IHC staining (Collagen type I, Collagen type III, Aggrecan, Versican, Decorin, Biglycan, Elastin, Fibrillin-1, Fibrillin-2), Collagen content (hydroxyproline), Tissue architecture, Cell morphology, Cell distribution, Cell arrangement, Vascularity, Inflammatory cell infiltration |  | Longitudinal: Proximal, Middle, Distal. |
| Spiesz et al. (2018) | Equine | **2T**  CDET, SDFT | **Collagen fibre organisation (birefringence)** | For both CDET and SDFT, IFM had lower total birefringence (lower collagen content/organisation) than FM. | IFM vs FM |
| Takahashi et al. (2018) | Bovine | **1T**  SDFT | Fibril diameter, **Cell density**, **Collagen-fibril density**, **GAG chain length, GAG chain angle** | Distal region had higher cell density than Middle and Proximal regions. Proximal region had higher cell density than Middle region.  Proximal region had higher fibril density than Middle and Distal regions.  Distal region had longer GAG chain length than Proximal region.  Distal region had larger GAG chain angle than Middle and Proximal regions. Middle region had larger GAG chain angle than Proximal region. | Longitudinal:  near-MTJ, Middle, near-TBI (Proximal, Middle, Distal) |
| Feo et al. (2019) | Avian (Pedrês chicken) | **1T**  SDFT | Cell density |  | Longitudinal: Proximal vs Distal |
| Zheng et al. (2024) | Avian (Turkey) | **1T**  Gastrocnemius (Achilles) | **Collagen structure organisation** | LM had greater organisation than EM and NM. EM additionally had greater organisation than NM. | Longitudinal:  Non- (NM; most distal), Late- (LM) and Early-mineralising (EM; most proximal) regions |

**Table S-2c.** Primary findings relating to differences in relation to age. Tendons/Ligaments or Outcomes of the study not relevant or statistically assessed for the detailed variable (*Age*) are greyed-out. Bold **outcomes** indicate those with statistically significant findings. *Refer to Table 5 in manuscript for acronym definitions.*

| Reference | Species | Tendons (T) / Ligaments (L) | | Outcomes | Major (statistically significant) findings | Age Cohorts |
| --- | --- | --- | --- | --- | --- | --- |
| Ingelmark (1948) | Murine (Rat) | **1T** | Achilles | ***Fibril diameter***, **Fibril cross striation length** | In OsO4 treated samples, Young (untrained) cohort had smaller fibril diameters than old (trained) cohort.  In OsO4 treated samples, Young (untrained) cohorts had narrower striation segments than the old (untrained) and old (trained) cohorts. | Young (untrained), Old (untrained) and Old (trained) |
| Wilmink et al. (1992) | Equine | **1T** | SDFT | **Crimp wavelength, Crimp angle** | In central region, Young animals had larger crimp angle than Old animals. In peripheral region, Young animals had larger crimp angle than Old animals.  In central region, Young animals had larger crimp wavelength than Old animals. In peripheral region, Young animals had larger crimp wavelength than Old animals. | Young (central and peripheral regions), Old (central and peripheral) |
| Patterson-Kane et al. (1997a) | Equine | **1T** | SDFT | *Fibril diameter*, *Crimp wavelength*, **Crimp angle** | For central region, the Old cohort has smaller crimp angle than the Young and Middle-Aged cohorts.  *[Additional pre-natal finding: Foetal cohort had larger crimp angle than all three adult groups (Young, Middle-Aged, Old)].*  *[Additional pre-natal finding: Foetal cohort had longer crimp wavelength than all three adult groups (Young, Middle-Aged, Old)].*  *[Additional pre-natal finding: Foetal cohort had smaller fibril diameter than young (central) cohort].* | Young (central and peripheral regions), Middle-Aged (central and peripheral), Old (central and peripheral)  *[Additional Foetal cohort (central region only)]* |
| Birch et al. (1999) | Equine | **2T** | DDFT, SDFT | Fibril diameter |  | Young, Old |
| Cook et al. (2004) | **Human** | **1T** | Patellar | Cell characteristics, Ground substance staining, Collagenous structure, Vascularity |  | Grouped according to “normal” vs “abnormal” score outcomes |
| Edwards et al. (2005) | Equine | **1T** | CDET | ***Fibril diameter****,* Collagen fibril density | The Young (control of short-term exercise) cohort had greater fibril diameter than the Old (control of long-term exercise) cohort. | Young, Old |
| Prado et al. (2006) | **Human** | **1T** | Tibialis Posterior | Vascular density |  | Young (under 40), Old (0ver 40) |
| Hadjicostas et al. (2007a) | **Human** | **2T** | Patellar, Quadriceps | Collagen-fibril density, Fibril diameter, Vascular density, Cell density |  | Single cohort, span 35 years |
| (Hadjicostas et al. 2007b) | **Human** | **3T** | Gracilis, Semitendinosus, Patellar | Collagen-fibril density, Fibril diameter, Vascular density, Cell density |  | Single cohort, span 44 years |
| Hadjicostas et al. (2008a) | **Human** | **4T**  **1L** | Gracilis, Semitendinosus, Patellar, Quadriceps  ACL | Collagen-fibril density, Fibril diameter, Vascular density, Cell density, Elastic fibril density, Collagen fibril-type density (Type I, Type III, Type V) |  | Single cohort, span 30 years |
| Hu et al. (2012) | Murine (Rat) | **1T** | *Unnamed* | **Collagen fibre orientation/orderliness, Fibre diameter** | The Old cohort had more orderly arranged fibre structure than the Young cohort.  The Old cohort had smaller *fibres* than the Young cohort. | Young, Old |
| Dunkman et al. (2013) | Murine (Mouse) | **1T** | Patellar | Fibril diameter, **Cell morphology**, **Cellularity**, **Collagen fibre alignment** | The Old cohort had lower cell density than the Young cohort.  The Young cohort had rounder cell morphology cell density than the Old cohort.  The Old cohort had more aligned fibres than the Young cohort. | Young, Old |
| Lavagnino et al. (2013) | Murine (Rat) | **1T** | Tail | **Cell density**, **Cell morphology**, αSMA presence | The Young cohort had lower cell density than the Mature and Middle-Aged cohort. The Mature cohort had lower cell density than the Middle-Aged cohort.  The Young cohort had rounder cell nuclei than the Mature and Middle-Aged cohort. The Mature cohort had rounder cell nuclei than the Middle-Aged cohort. | Young (1-month), Mature (3-month), Middle-Aged (12-month) |
| Thorpe et al. (2013) | Equine | **1T** | SDFT | IFM area | Young SDFTs had greater IFM area percentage than Old SDFTs. | Young, Old |
| Legerlotz et al. (2014) | Murine (Mouse) | **1T** | Tail | **Crimp wavelength**, **Crimp height, Crimp angle**, Crimp planarity**, Cell length, Cell gap width (cell spacing),** **Cell density, Cell track distance (distribution)** | From both two-photon and brightfield microscopy, the 3w cohort had shorter crimp wavelength than all other cohorts (6w, 9w, 12w, 6m). For two-photon microscopy, 6w and 6m cohorts had shorter crimp wavelength than the 9w cohort.  The 3w cohort had shorter crimp height than the 6w and 9w cohorts.  The 6m cohort had smaller crimp angle than the 6w and 9w cohorts.  The 3w cohort had shorter cell length than the 9w, 12w and 6m cohorts. The 6w cohort had shorter cell length than the 12w cohort.  The 6m cohort had larger gap width than all other cohorts (3w, 6w, 9w, 12w).  The 3w cohort had larger cell density than all other cohorts (6w, 9w, 12w, 6m). The 6w cohort had smaller cell density than the 12w and 6m cohorts.  The 3w cohort had smaller cell track distance than the 6w and 9w cohorts. | 3-weeks, 6-weeks, 9-weeks, 12-weeks and 6-months |
| Tilley et al. (2014) | **Human** | **2T** | Subscapularis, Supraspinatus | **Fibril diameter,** Fibril angle (orientation), Crimp wavelength, **Fibre-thickness ratios (PSR staining)** | Supraspinatus tendon (young cohort) had thinner fibril diameters than Subscapularis tendon (old cohort).  Supraspinatus tendon (young cohort) had lower ratio of thick-to-thin diameter fibrils than Subscapularis tendon (old cohort). | Young (Supraspinatus), Old (Subscapularis) |
| Thorpe et al. (2016b) | Equine | **1T** | SDFT | Cell density |  | Young, Old |
| Godinho et al. (2017) | Equine | **2T** | CDET, SDFT | **Elastin content (IHC staining), IFM:FM area ratio,** **Elastin fibre orientation/alignment** | For IFM, elastin content increased with age in the SDFT. For FM, elastin content decreased with age in the CDET.  For both SDFT and CDET, IFM:FM ratio increased with age.  In SDFT, the Old cohort had more randomly distributed elastin fibres than the Young cohort. Additionally, for SDFT, the Old cohort had less overall organisation than the Young cohort. | Young, Old |
| Marquez-Arabia et al. (2017) | **Human** | **1T** | Gluteus Medius | **Vascular density** | Vascular density was correlated with age; *convex asymmetrical quadratic correlation: density increased with age (18 to approx. 40 years) and then decreased with further aging (over 50 years).* | Single cohort, span 50 years |
| Pardes et al. (2017) | Murine (Rat) | **1T** | Achilles | **Cell morphology**, **Cell density**, Proteoglycan content (Safranin-O/Fast Green staining) | The Old cohort had smaller dell density than Middle-Aged cohort.  The Old cohort had more rounded cell morphology than the Middle-Aged and Young cohorts. | Young, Middle-Aged, Old |
| Gagliano et al. (2018) | **Human** | **2T** | Gracilis, Semitendinosus | GAG/Proteoglycan staining intensity (alcian-blue), Sirius red staining intensity (fibrillary-collagen) |  | Young, Old |
| Shu et al. (2018) | Murine (Mouse) | **2T** | Achilles, Tail | **Fibril diameter** | For Both Tail and Achilles Tendons, the Old cohort had larger fibril diameter than the Young cohort. | Young, Old |
| Spiesz et al. (2018) | Equine | **2T** | CDET, SDFT | Collagen fibre organisation (birefringence), Crimp angle, Crimp wavelength |  | Young, Old |
| Feo et al. (2019) | Avian (Pedrês chicken) | **1T** | SDFT | **Cell density** | In both regions, the Young cohort had greater cell density than the Middle and Old cohorts. | Younger (1-month), Middle (5-months), Older (8-months) |
| Hayashi et al. (2019) | Canine | **1T**  **3L** | LDET  CaCL, CrCL, MCL | **Cell morphology, Cell density** | In CaCL, Cell density decreased with age.  In CaCL, Fusiform cell density decreased with age. | Single cohort (canine), span 14 years |
| Asai et al. (2022) | **Human** | **1T** | Semitendinosus | **Cell morphology**, **Cell density,** **Fibril diameter (and distribution)** | The Immature cohort had greater cell major-to-minor-length ratio (rounder cell morphology) than the Young and Adult cohorts. The Young cohort had greater cell major-to-minor-length ratio than the Adult cohort.  The Immature cohort had greater cell density than the Young and Adult cohorts. The Young cohort had greater cell density than the Adult cohort.  For mean fibril diameter, the Adult cohort had larger mean diameters then the Immature cohort. For 20^th^ percentile, the Adult cohort had larger diameters than the Young and Immature cohorts. For 40^th^ percentile, the Adult cohort had larger diameters than the Immature cohort. For 60^th^ percentile, the Adult cohort had larger diameters than the Immature cohort. For 80^th^ percentile, the Adult cohort had larger diameters than the Immature cohort.  Additionally, fibril diameter distribution differed between all cohorts, with histograms shifted from right skewed in the Immature cohort (predominantly small-diameter fibrils) to flatter in the Adult cohort (more even spread with more larger-diameter fibrils). | Immature, Young, Adult |
| Naot et al. (2022) | **Human** | **1T** | Semitendinosus | Fibril diameter |  | Young (under 20), Old (over 20) |
| Fjordbakk and Marques-Smith (2023) | Equine | **3T** | Medial Patellar, Intermediate Patellar, Lateral Patellar | ***IFM thickness, IFM vascularity,*** **Adipose Infiltration** | Interfascicular endotenon (IFM) characteristics had significant correlation with age for Medial and Intermediate Patellar ligaments (‘tendons’). *[For both Medial and Lateral ligaments, age increased IFM size (distinction) and vasculature (prominence)].*  Fatty infiltration increased with age for Medial and Intermediate ligaments. | Single cohort (equine), span 17 years. |
| Johnson et al. (2023) | **Human** | **4T** | Infraspinatus, Subscapularis, Supraspinatus, Teres Minor | Collagen organisation, Cell appearance, Vascular presence, Collagenous architecture, GAG/Proteoglycan/ground substance presence, **Total histomophological score** | For Infraspinatus tendon, the Aged cohort had greater total histomorphological (Bonar) score than the Younger cohort. | Younger (< 36 years), Aged (> 55 years) |
| Tinguely et al. (2023) | **Murine (Rat), Human** | **2T** | Achilles [rat; human], Patellar [rat] | Cellularity, Segmented nuclei count per image, **Nuclear aspect ratio,** **individual shape mode fractions**, Proteoglycan richness (SAFO), Presence of CD31 (endothelial cells), presence of αSMA (myofibroblasts), and presence of CD146 (tendon stem/progenitor cells). | Cell morphology (nuclear aspect ratio) was correlated with age.  The 1m cohort had different cell shape distributions than the 8m, 19m and 28m cohorts. The 8m and 19m cohorts had different cell shape distributions than the 29m cohort.  Age had an effect on proteoglycan richness associated cell morphology. | 1-month, 8-months, 19-months, 28-months |

**Table S-2d.** Primary findings relating to differences between males and females. Tendons/Ligaments or Outcomes of the study not relevant or statistically assessed for the detailed variable (*Sex*) are greyed-out. Bold **outcomes** indicate those with statistically significant findings. *Refer to Table 5in manuscript for acronym definitions.*

| Reference | Species | Tendons (T) / Ligaments (L) | | Outcomes | Major (statistically significant) findings |
| --- | --- | --- | --- | --- | --- |
| Prado et al. (2006) | **Human** | **1T** | Tibialis Posterior | Vascular density |  |
| Hadjicostas et al. (2007a) | **Human** | **2T** | Patellar, Quadriceps | Collagen-fibril density, Fibril diameter, Vascular density, Cell density |  |
| Hadjicostas et al. (2007b) | **Human** | **3T** | Gracilis, Semitendinosus, Patellar | Collagen-fibril density, Fibril diameter, Vascular density, Cell density |  |
| Hadjicostas et al. (2008a) | **Human** | **4T**  **1L** | Gracilis, Semitendinosus, Patellar, Quadriceps  ACL | Collagen-fibril density, Fibril diameter, Vascular density, Cell density, Elastic fibril density, Collagen fibril-type density (Type I, Type III, Type V) |  |
| Pardes et al. (2016) | Murine (Rat) | **1T** | Achilles | Cell morphology, Cellularity, Proteoglycan content |  |
| Sarver et al. (2017) | Murine (Mouse) | **1T** | Achilles | **Cell density** | Females had larger cell density than Males. |
| Hayashi et al. (2019) | Canine | **1T**  **3L** | LDET  CaCL, CrCL, MCL | Cell morphology (fusiform density, ovoid density, spheroid density), Cell density |  |
| Asai et al. (2022) | **Human** | **1T** | Semitendinosus | Cell morphology, Cell density, Fibril diameter |  |

**Table S-2e.** Primary findings relating to differences in relation to *other* variables. Tendons/Ligaments or Outcomes of the study not relevant or statistically assessed for the detailed variable (*Other*) are greyed-out. Bold **outcomes** indicate those with statistically significant findings. *Refer to Table 5in manuscript for acronym definitions.*

| Reference | Species | Tendons (T) / Ligaments (L) | | Outcomes | Major (statistically significant) findings | Variable | Variable specifics/cohorts |
| --- | --- | --- | --- | --- | --- | --- | --- |
| Ingelmark (1948) | Murine (Rat) | **1T** | Achilles | **Fibril diameter,** Fibril cross striation length | In OsO4 treated fibrils of the old cohorts, the Trained cohort had larger fibril diameters than the Untrained cohort. | Lifestyle – Physical | Effect of exercise regime:  Trained vs Untrained |
| Odetti et al. (2000) | Murine (Rat) | **1T** | Tail | **Fibril diameter,** Fibril D-band interval, **Fibril D-band cleft depth** | The Diabetic cohort had larger fibril diameters than the Non-Diabetic cohort.  The Diabetic cohort had larger D-Band cleft depth than the Non-Diabetic cohort. | Comorbidities – Diabetes | Effect of diabetes status:  Diabetic vs Non-Diabetic |
| Cook et al. (2004) | **Human** | **1T** | Patellar | Cell characteristics, Ground substance staining, Collagenous structure, Vascularity |  | Demographics  Lifestyle – Physical  Comorbidities – ACL Injury | Correlations made between scored outcomes (normal VS abnormal result) versus anthropometric measures of weight, height, hours per week of activity, years sport, and time from ACL rupture to reconstruction with the studied tendon |
| Edwards et al. (2005) | Equine | **1T** | CDET | ***Fibril diameter,*** Collagen fibril density | ******** The Short-Term Exercise (younger) cohort had greater fibril diameters than the Long-Term Exercise (older) cohort.  ***Note: differences likely resulting from confounding factor of Age as an additional variable* | Lifestyle – Physical | Effect of exercise regime:  Short-Term (younger cohort) vs Long-Term (older cohort) exercise regime, and respective controls |
| Prado et al. (2006) | **Human** | **1T** | Tibialis Posterior | Vascular density |  | Anatomical | Comparison between left- and right-limb |
| Funakoshi et al. (2008) | Caprine | **2T** | Infraspinatus, Patellar | **Fascicle diameter, Crimp wavelength** | Individual animal had an effect on fascicle diameter.  Individual animal had an effect on fascicle diameter. | Individual Specifics | Effect of individual animal on outcome |
| Stanley et al. (2008) | Equine | **2T** | CDET, SDFT | Cell density, **Cell morphology** | For all tendons combined (CDET, central SDFT, peripheral SDFT):  The Long-Term Exercise cohort had lower proportion of type 1 (spindle) cells than the respective Control tendons. | Lifestyle – Physical | Effect of exercise regime:  Long-Term, Short-Term, and Foal-Trained exercise regimes, and respective controls |
| Hansen et al. (2009) | **Human** | **1T** | Patellar | Fibril density, Fibril-occupied area**, Fibril diameter** | ERT-use (high estrogen) cohort had greater percentage of medium (60-90nm) fibrils than the Control (low estrogen) cohort.  Control cohort had a higher percentage of large (>90nm) fibrils than ERT cohort. | Biological | Comparison between different hormonal levels/interventions in post-menopausal women in response to exercise:  Estrogen replacement therapy (ERT) (following hysterectomy) vs postmenopausal control |
| Frizziero et al. (2011) | Murine (Rat) | **1T** | Patellar | **Proteoglycan (PG) content, Collagen fibre organisation/alignment** | ** Trained tendons had higher PG staining presence than Untrained and Detrained tendons.  ***Note: PG content scoring was specific for tendon insertion sight (enthesis).*  Trained tendons had more organised fibre organisation/alignment than Untrained and Detrained tendons. | Lifestyle – Physical | Comparison between different exercise regimes:  Untrained, Trained, Detrained |
| Lenskjold et al. (2015) | **Human** | **1T** | Achilles | Fibril density, Fibril diameter, Fibril area |  | Lifestyle - Physical | Effects of activity levels during youth:  Athletes with prior high physical activity (HAY) versus low physical activity (LAY) during their youth |
| (Pardes et al. 2016) | Murine (Rat) | **1T** | Achilles | Cell morphology, Cellularity, Proteoglycan content |  | Biological | Effects of sex-hormonal level (female vs ovariectomised female) |
| Mazon et al. (2018) | Murine (Rat) | **1T** | Calcaneal (Achilles) | **Collagen organisation (birefringence properties)** | Strength Trained tendons had lower birefringence (less organised/aligned structure) than Untrained, Hypertrophy Trained and Resistance Trained tendons. | Lifestyle – Physical | Effect of exercise regime:  Untrained, Resistance Trained, Hypertrophy Trained, Strength Trained |
| Hayashi et al. (2019) | Canine | **1T**  **3L** | LDET  CaCL, CrCL, MCL | Cell morphology (fusiform density, ovoid density, spheroid density), Cell density |  | Demographics | Effects of body weight |
| Asai et al. (2022) | **Human** | **1T** | Semitendinosus | Cell morphology, Cell density, Fibril diameter |  | Demographics | Correlation between cell density and weight, height and BMI in Young and Adult cohorts |
| Bolam et al. (2022) | Murine (Rat) | **1T** | Achilles | Cell density, Cell morphology, Collagen fibre alignment, Fibril diameter |  | Lifestyle – Diet | Effect of diet:  Low-Fat (control), High-Fat, High-then-Low-Fat diet regimes |
| Gsell et al. (2023) | **Bovine, Murine (Rat)** | **3T** | LDET [bovine], SDFT [bovine], Tail [rat] | **Fibril CSA, Fibril Aspect Ratio** | Rat Tail fibrils had larger CSA (diameter) than Bovine LDET fibrils.  Rat Tail fibrils had larger Aspect Ratio (aka rounder/plumper) than Bovine LDET fibrils. | Species** | Comparison between species.  ***Note: different anatomical tendons are used between species* |

**REFFRENCES**

Asai K., Nakase J., Ishikawa T., Yoshimizu R., Kimura M., Ozaki N. and Tsuchiya H. (2022). Differences in cellular and microstructural properties of the semitendinosus muscle tendon between young and adult patients, *Journal of Orthopaedic Science,* 27(2): 478-485.

Biasutti S., Dart A., Smith M., Blaker C., Clarke E., Jeffcott L. and Little C. (2017). Spatiotemporal variations in gene expression, histology and biomechanics in an ovine model of tendinopathy, *PloS One,* 12(10): e0185282.

Birch H.L., Bailey J.V.B., Bailey A.J. and Goodship A.E. (1999). Age‐related changes to the molecular and cellular components of equine flexor tendons, *Equine Veterinary Journal,* 31(5): 391-396.

Bolam S.M., Konar S., Park Y.E., Callon K.E., Workman J., Monk A.P., . . . Musson D.S. (2022). A high-fat diet has negative effects on tendon resident cells in an in vivo rat model, *International Orthopaedics,* 46(5): 1181-1190.

Cook J.L., Feller J.A., Bonar S.F. and Khan K.M. (2004). Abnormal tenocyte morphology is more prevalent than collagen disruption in asymptomatic athletes' patellar tendons, *Journal of Orthopaedic Research,* 22(2): 334-8.

Dunkman A.A., Buckley M.R., Mienaltowski M.J., Adams S.M., Thomas S.J., Satchell L., . . . Soslowsky L.J. (2013). Decorin expression is important for age-related changes in tendon structure and mechanical properties, *Matrix Biology,* 32(1): 3-13.

Edwards L.J., Goodship A.E., Birch H.L. and Patterson-Kane J.C. (2005). Effect of exercise on age-related changes in collagen fibril diameter distributions in the common digital extensor tendons of young horses, *American Journal of Veterinary Research,* 66(4): 564-8.

Feo H.B., Biancalana A., Romero Nakagaki W., Aparecida De Aro A. and Gomes L. (2019). Morphological Alterations and Increased Gelatinase Activity in the Superficial Digital Flexor Tendon of Chickens During Growth and Maturation, *Anatomical Record (Hoboken),* 302(6): 964-972.

Fjordbakk C.T. and Marques-Smith P. (2023). The equine patellar ligaments and the infrapatellar fat pad - a microanatomical study, *BMC Veterinary Research,* 19(1): 20.

Franchi M., Quaranta M., Macciocca M., De Pasquale V., Ottani V. and Ruggeri A. (2009). Structure relates to elastic recoil and functional role in quadriceps tendon and patellar ligament, *Micron,* 40(3): 370-7.

Frizziero A., Fini M., Salamanna F., Veicsteinas A., Maffulli N. and Marini M. (2011). Effect of training and sudden detraining on the patellar tendon and its enthesis in rats, *BMC Musculoskeletal Disorders,* 12: 20.

Funakoshi T., Schmid T., Hsu H.P. and Spector M. (2008). Lubricin distribution in the goat infraspinatus tendon: a basis for interfascicular lubrication, *Journal of Bone and Joint Surgery (American Volume),* 90(4): 803-14.

Gagliano N., Menon A., Cabitza F., Compagnoni R. and Randelli P. (2018). Morphological and molecular characterization of human hamstrings shows that tendon features are not influenced by donor age, *Knee Surgery, Sports Traumatology, Arthroscopy,* 26(1): 343-352.

Gibson K.T., Burbidge H.M. and Robertson I.D. (2002). The effects of polyester (terylene) fibre implants on normal equine superficial digital flexor tendon, *New Zealand Veterinary Journal,* 50(5): 186-94.

Godinho M.S.C., Thorpe C.T., Greenwald S.E. and Screen H.R.C. (2017). Elastin is Localised to the Interfascicular Matrix of Energy Storing Tendons and Becomes Increasingly Disorganised With Ageing, *Scientific Reports,* 7(1): 9713.

Gsell K.Y., Veres S.P. and Kreplak L. (2023). Single collagen fibrils isolated from high stress and low stress tendons show differing susceptibility to enzymatic degradation by the interstitial collagenase matrix metalloproteinase-1 (MMP-1), *Matrix Biol Plus,* 18: 100129.

Hadjicostas P.T., Soucacos P.N., Berger I., Koleganova N. and Paessler H.H. (2007a). Comparative analysis of the morphologic structure of quadriceps and patellar tendon: a descriptive laboratory study, *Arthroscopy : the journal of arthroscopic & related surgery : official publication of the Arthroscopy Association of North America and the International Arthroscopy Association,* 23(7): 744-750.

Hadjicostas P.T., Soucacos P.N., Koleganova N., Krohmer G. and Berger I. (2008a). Comparative and morphological analysis of commonly used autografts for anterior cruciate ligament reconstruction with the native ACL: an electron, microscopic and morphologic study, *Knee Surgery, Sports Traumatology, Arthroscopy,* 16(12): 1099-107.

Hadjicostas P.T., Soucacos P.N., Koleganova N., Piecha G., Krohmer G. and Berger I. (2008b). Comparative analysis of the microstructure of the hamstring tendons: an electron microscopic, histologic, and morphologic study, *Journal of Surgical Orthopaedic Advances,* 17(3): 153-8.

Hadjicostas P.T., Soucacos P.N., Paessler H.H., Koleganova N. and Berger I. (2007b). Morphologic and histologic comparison between the patella and hamstring tendons grafts: a descriptive and anatomic study, *Arthroscopy : the journal of arthroscopic & related surgery : official publication of the Arthroscopy Association of North America and the International Arthroscopy Association,* 23(7): 751-756.

Hansen M., Kongsgaard M., Holm L., Skovgaard D., Magnusson S.P., Qvortrup K., . . . Kjaer M. (2009). Effect of estrogen on tendon collagen synthesis, tendon structural characteristics, and biomechanical properties in postmenopausal women, *Journal of Applied Physiology (1985),* 106(4): 1385-93.

Hansen P., Haraldsson B.T., Aagaard P., Kovanen V., Avery N.C., Qvortrup K., . . . Peter Magnusson S. (2010). Lower strength of the human posterior patellar tendon seems unrelated to mature collagen cross-linking and fibril morphology, *Journal of Applied Physiology (1985),* 108(1): 47-52.

Hayashi K., Bhandal J., Kim S.Y., Walsh N., Entwistle R., Stover S.M. and Kapatkin A.S. (2019). Comparative histomorphometric analysis of cellular phenotype in canine stifle ligaments and tendon, *Veterinary Surgery,* 48(6): 1013-1018.

Herod T.W., Chambers N.C. and Veres S.P. (2016). Collagen fibrils in functionally distinct tendons have differing structural responses to tendon rupture and fatigue loading, *Acta Biomaterialia,* 42: 296-307.

Hosaka Y.Z., Takahashi H., Uratsuji T., Tangkawattana P., Ueda H. and Takehana K. (2010). Comparative study of the characteristics and properties of tendinocytes derived from three tendons in the equine forelimb, *Tissue and Cell,* 42(1): 9-17.

Hu W., Li H., Wang C., Gou S. and Fu L. (2012). Characterization of collagen fibers by means of texture analysis of second harmonic generation images using orientation-dependent gray level co-occurrence matrix method, *Journal of Biomedical Optics,* 17(2): 026007.

Ingelmark B.E. (1948). The Structure of Tendons at Various Ages and Under Different Functional Conditions. II, *Cells Tissues Organs,* 6(3): 193-225.

Johnson J., Von Stade D., Gadomski B., Regan D., Easley J., Sikes K.J., . . . Mcgilvray K. (2023). Biomechanical and histological changes secondary to aging in the human rotator cuff: A preliminary analysis, *Journal of Orthopaedic Research,* 41(10): 2221-2231.

Kharaz Y.A., Canty-Laird E.G., Tew S.R. and Comerford E.J. (2018). Variations in internal structure, composition and protein distribution between intra- and extra-articular knee ligaments and tendons, *Journal of Anatomy,* 232(6): 943-955.

Lavagnino M., Gardner K. and Arnoczky S.P. (2013). Age-related changes in the cellular, mechanical, and contractile properties of rat tail tendons, *Connective Tissue Research,* 54(1): 70-5.

Legerlotz K., Dorn J., Richter J., Rausch M. and Leupin O. (2014). Age-dependent regulation of tendon crimp structure, cell length and gap width with strain, *Acta Biomaterialia,* 10(10): 4447-55.

Lenskjold A., Kongsgaard M., Larsen J.O., Nielsen R.H., Kovanen V., Aagaard P., . . . Magnusson S.P. (2015). The influence of physical activity during youth on structural and functional properties of the Achilles tendon, *Scandinavian Journal of Medicine and Science in Sports,* 25(1): 25-31.

Marquez-Arabia W.H., Gomez-Hoyos J., Gomez M., Florez I., Gallo J.A., Monsalve F., . . . Martin H.D. (2017). Influence of Aging on Microvascular Supply of the Gluteus Medius Tendon: A Cadaveric and Histologic Study, *Arthroscopy,* 33(7): 1354-1360.

Mazon J., De Aro A.A., Simoes P.W. and Pimentel E.R. (2018). Effect of different resistance-training protocols on the extracellular matrix of the calcaneal tendon of rats, *Ann Anat,* 216: 75-81.

Mazzocca A.D., Mccarthy M.B.R., Ledgard F.A., Chowaniec D.M., Mckinnon W.J., Delaronde S., . . . Beitzel K. (2013). Histomorphologic Changes of the Long Head of the Biceps Tendon in Common Shoulder Pathologies, *Arthroscopy,* 29(6): 972-981.

Naot D., Choi A.J., Street M., Pronk M., Zhao A., Thambyah A., . . . Musson D.S. (2022). Age-related differences in hamstring tendon used as autograft in reconstructive anterior cruciate ligament surgery, *International Orthopaedics,* 46(4): 845-853.

Odetti P., Aragno I., Rolandi R., Garibaldi S., Valentini S., Cosso L., . . . Marinari U.M. (2000). Scanning force microscopy reveals structural alterations in diabetic rat collagen fibrils: role of protein glycation, *Diabetes/Metabolism Research and Reviews,* 16(2): 74-81.

Pardes A.M., Beach Z.M., Raja H., Rodriguez A.B., Freedman B.R. and Soslowsky L.J. (2017). Aging leads to inferior Achilles tendon mechanics and altered ankle function in rodents, *Journal of Biomechanics,* 60: 30-38.

Pardes A.M., Freedman B.R., Fryhofer G.W., Salka N.S., Bhatt P.R. and Soslowsky L.J. (2016). Males have Inferior Achilles Tendon Material Properties Compared to Females in a Rodent Model, *Annals of Biomedical Engineering,* 44(10): 2901-2910.

Patterson-Kane J.C., Firth E.C., Goodship A.E. and Parry D.A. (1997a). Age-related differences in collagen crimp patterns in the superficial digital flexor tendon core region of untrained horses, *Australian Veterinary Journal,* 75(1): 39-44.

Patterson-Kane J.C., Parry D.A., Goodship A.E. and Firth E.C. (1997b). Exercise modifies the age-related change in crimp pattern in the core region of the equine superficial digital flexor tendon, *New Zealand Veterinary Journal,* 45(4): 135-9.

Prado M.P., De Carvalho A.E., Jr., Rodrigues C.J., Fernandes T.D., Mendes A.A. and Salomao O. (2006). Vascular density of the posterior tibial tendon: a cadaver study, *Foot and Ankle International,* 27(8): 628-31.

Ristaniemi A., Regmi D., Mondal D., Torniainen J., Tanska P., Stenroth L., . . . Korhonen R.K. (2021). Structure, composition and fibril-reinforced poroviscoelastic properties of bovine knee ligaments and patellar tendon, *Journal of the Royal Society Interface,* 18(174): 20200737.

Sarver D.C., Kharaz Y.A., Sugg K.B., Gumucio J.P., Comerford E. and Mendias C.L. (2017). Sex differences in tendon structure and function, *Journal of Orthopaedic Research,* 35(10): 2117-2126.

Shu C.C., Smith M.M., Appleyard R.C., Little C.B. and Melrose J. (2018). Achilles and tail tendons of perlecan exon 3 null heparan sulphate deficient mice display surprising improvement in tendon tensile properties and altered collagen fibril organisation compared to C57BL/6 wild type mice, *PeerJ,* 6: e5120.

Spiesz E.M., Thorpe C.T., Thurner P.J. and Screen H.R.C. (2018). Structure and collagen crimp patterns of functionally distinct equine tendons, revealed by quantitative polarised light microscopy (qPLM), *Acta Biomaterialia,* 70: 281-292.

Stanley R.L., Goodship A.E., Edwards B., Firth E.C. and Patterson-Kane J.C. (2008). Effects of exercise on tenocyte cellularity and tenocyte nuclear morphology in immature and mature equine digital tendons, *Equine Veterinary Journal,* 40(2): 141-6.

Takahashi N., Hirose T., Minaguchi J.A., Ueda H., Tangkawattana P. and Takehana K. (2018). Fibrillar architecture at three different sites of the bovine superficial digital flexor tendon, *Journal of Veterinary Medical Science,* 80(3): 405-412.

Thorpe C.T., Karunaseelan K.J., Ng Chieng Hin J., Riley G.P., Birch H.L., Clegg P.D. and Screen H.R. (2016a). Distribution of proteins within different compartments of tendon varies according to tendon type, *Journal of Anatomy,* 229(3): 450-8.

Thorpe C.T., Peffers M.J., Simpson D., Halliwell E., Screen H.R. and Clegg P.D. (2016b). Anatomical heterogeneity of tendon: Fascicular and interfascicular tendon compartments have distinct proteomic composition, *Scientific Reports,* 6: 20455.

Thorpe C.T., Udeze C.P., Birch H.L., Clegg P.D. and Screen H.R. (2013). Capacity for sliding between tendon fascicles decreases with ageing in injury prone equine tendons: a possible mechanism for age-related tendinopathy?, *European Cells & Materials,* 25: 48-60.

Tilley J.M., Murphy R.J., Chaudhury S., Czernuszka J.T. and Carr A.J. (2014). Effect of tear size, corticosteroids and subacromial decompression surgery on the hierarchical structural properties of torn supraspinatus tendons, *Bone Joint Res,* 3(8): 252-61.

Tinguely Y., Shi V., Klatte-Schulz F., Duda G.N., Freedman B.R. and Mooney D.J. (2023). Aging and injury affect nuclear shape heterogeneity in tendon, *Journal of Orthopaedic Research,* 41(10): 2186-2194.

Watanabe T., Hosaka Y., Yamamoto E., Ueda H., Sugawara K., Takahashi H. and Takehana K. (2005). Control of the collagen fibril diameter in the equine superficial digital flexor tendon in horses by decorin, *Journal of Veterinary Medical Science,* 67(9): 855-60.

Weiss M., Unterhauser F.N. and Weiler A. (2012). Crimp frequency is strongly correlated to myofibroblast density in the human anterior cruciate ligament and its autologous tendon grafts, *Knee Surgery, Sports Traumatology, Arthroscopy,* 20(5): 889-95.

Williams L.N., Elder S.H., Horstemeyer M.F. and Harbarger D. (2008). Variation of diameter distribution, number density, and area fraction of fibrils within five areas of the rabbit patellar tendon, *Ann Anat,* 190(5): 442-51.

Wilmink J., Wilson A.M. and Goodship A.E. (1992). Functional significance of the morphology and micromechanics of collagen fibres in relation to partial rupture of the superficial digital flexor tendon in racehorses, *Research in Veterinary Science,* 53(3): 354-359.

Zheng K., Zhong J., Hu J., Nebbiolo E., Sanchez-Weatherby J., Tang T., . . . Bell J. (2024). Effects of mineralization on the hierarchical organization of collagen-a synchrotron X-ray scattering and polarized second harmonic generation study, *Interface Focus,* 14(3): 20230046.

Zhu J., Zhang X., Ma Y., Zhou C. and Ao Y. (2012). Ultrastructural and morphological characteristics of human anterior cruciate ligament and hamstring tendons, *Anatomical Record (Hoboken),* 295(9): 1430-6.
